# Exceptional dynamical quantum phase transitions in periodically driven systems

**DOI:** 10.1038/s41467-021-25355-3

**Published:** 2021-09-01

**Authors:** Ryusuke Hamazaki

**Affiliations:** Nonequilibrium Quantum Statistical Mechanics RIKEN Hakubi Research Team, RIKEN Cluster for Pioneering Research (CPR), RIKEN iTHEMS, Wako, Saitama, Japan

**Keywords:** Quantum physics, Phase transitions and critical phenomena, Statistical physics

## Abstract

Extending notions of phase transitions to nonequilibrium realm is a fundamental problem for statistical mechanics. While it was discovered that critical transitions occur even for transient states before relaxation as the singularity of a dynamical version of free energy, their nature is yet to be elusive. Here, we show that spontaneous symmetry breaking can occur at a short-time regime and causes universal dynamical quantum phase transitions in periodically driven unitary dynamics. Unlike conventional phase transitions, the relevant symmetry is antiunitary: its breaking is accompanied by a many-body exceptional point of a nonunitary operator obtained by space-time duality. Using a stroboscopic Ising model, we demonstrate the existence of distinct phases and unconventional singularity of dynamical free energy, whose signature can be accessed through quasilocal operators. Our results open up research for hitherto unknown phases in short-time regimes, where time serves as another pivotal parameter, with their hidden connection to nonunitary physics.

## Abstract (web summary)

Understanding phase transitions in systems out of equilibrium is a topic of high interest. Here the author discusses the spontaneous antiunitary symmetry breaking leading to exceptional dynamical quantum phase transitions in driven many-body systems.

## Introduction

Phase transition^1,2^ is one of the most fundamental collective phenomena in macroscopic systems. Recent experiments on artificial quantum many-body systems motivate researchers to understand phases and their transitions in systems out of equilibrium. Various nonequilibrium phases are proposed including e.g., many-body localized phases^3,4^, Floquet topological phases^5,6^, and discrete-time crystals^7–9^.

Recently, dynamical quantum phase transitions (DQPTs) particularly gather great attention as a nonequilibrium counterpart of equilibrium phase transition, which occurs for transient times of quantum relaxation^10,11^. Defined as the singularity of the so-called dynamical free energy (especially at critical times), which is calculated from the overlap between the time-evolved and reference states, the DQPT has been actively studied theoretically^12–25^ and experimentally^26,27^.

Despite extensive studies, the nature of DQPTs is yet to be elusive. One of the important problems is what mechanism leads to DQPTs. Several studies find that some DQPTs are associated with equilibrium/steady-state phase transition^13,22^. On the other hand, DQPTs without such relations may also exist^14,20^, which indicates that DQPTs can be caused by an unconventional mechanism unique to the finite-time (high-frequency) regime of quantum relaxation. Another open problem is the universality and criticality of DQPTs. Although typical DQPTs are accompanied by cusps of dynamical free energies^10,11^, several works report DQPTs with different types of singularities^16,23^. However, a clear understanding of the universality and criticality of DQPTs is far from complete.

**Fig. 1:**
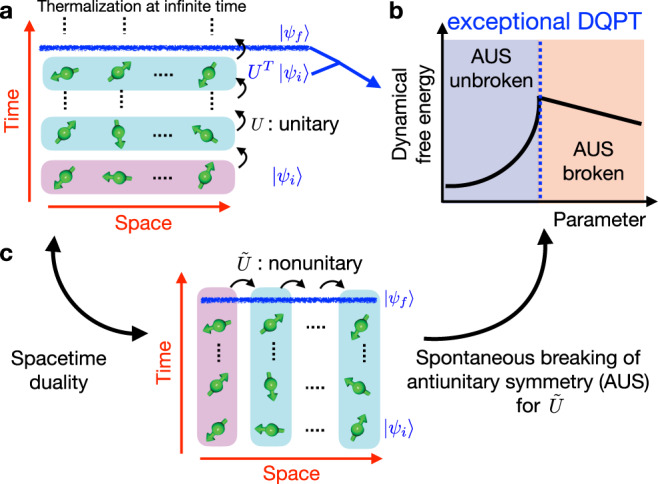
Schematic of the exceptional dynamical quantum phase transition (DQPT). **a** Periodically driven unitary dynamics described by an operator *U*. While our nonintegrable system thermalizes for local observables at infinite times, we here discuss the phase transitions occurring at finite times. **b** Example of the exceptional DQPT. Dynamical free energy, which is obtained from the overlap between the time-evolved state ${U}^{T}|{\psi }_{i}\rangle$ and the reference state $|{\psi }_{f}\rangle$, has a divergent derivative at some critical parameter approached from the antiunitary-symmetry (AUS) unbroken phase. **c** Origin of the exceptional DQPT as the spontaneous AUS breaking. Using the spacetime duality, we find a hidden nonunitary transfer operator $\tilde{U}$ that propagates in space direction. We uncover that the exceptional DQPTs arise when the AUS for $\tilde{U}$ is spontaneously broken.

In this work, we find universal DQPTs in periodically driven unitary dynamics caused by the spontaneous antiunitary symmetry (AUS) breaking. While spontaneous symmetry breaking is a fundamental mechanism for conventional phase transitions, several distinct features appear in our results. First, the AUS breaking in our model occurs uniquely at finite times and cannot be captured by conventional equilibrium or steady-state phases. Second, the AUS appears as a symmetry of a hidden nonunitary transfer operator, which is obtained by switching the role of space and time. Consequently, the universality and criticality found in the unitary dynamics are characterized by those of the exceptional point, which recently gathers great attention in non-Hermitian physics^28,29^; thus we call the transition the exceptional DQPT. To demonstrate our discovery, we particularly use a stroboscopic chaotic Ising chain and show that the derivative of dynamical free energy defined at finite times can diverge through changes of a parameter (Fig. 1a, b). Using the recently developed technique called the spacetime duality^30–33^ and determining the hidden nonunitary operator, we discuss several properties of the exceptional DQPT besides the divergence of the dynamical free energy (Fig. 1c). For example, instead of the long-range order associated with conventional symmetry breaking, we show that the generalized correlation function has the divergent correlation length at transition and exhibits oscillatory long-range order after antiunitary symmetry breaking. Finally, we demonstrate that the signatures of the exceptional DQPTs are observed through quasi-local observables that are accessible by state-of-the-art experiments^8,34^. Notably, we argue that the signature of the exceptional DQPTs is easier to observe than that of the normal DQPTs because of their strong singularity. Our results make an important step toward understanding the nature of phase transitions occurring in a short-time regime, which goes beyond conventional phase transitions since time serves as another crucial parameter here, with their hidden connection to nonunitary physics.

## Results

### Stroboscopic Ising chains and dynamical free energy

To demonstrate our finding, we introduce a one-dimensional quantum stroboscopic spin model^30,31,35^ composed of Ising interaction and subsequent global rotation. This model is a prototypical model for quantum chaotic dynamics and can be realized in experiments of e.g., trapped ions^8^. Its unitary time evolution for a single step can be written as

1 $$U={{{{{{\mathrm{e}}}}}}}^{{{{{{\mathrm{-i}}}}}}\mathop{\sum }\nolimits_{j = 1}^{L}b{\sigma }_{j}^{x}}{{{{{{\mathrm{e}}}}}}}^{{{{{{\mathrm{-i}}}}}}\mathop{\sum }\nolimits_{j = 1}^{L}J{\sigma }_{j}^{z}{\sigma }_{j+1}^{z}{{{{{\mathrm{-i}}}}}}\mathop{\sum }\nolimits_{j = 1}^{L}h{\sigma }_{j}^{z}},$$

where we impose a periodic boundary condition.

Let us consider a time-evolved state ${U}^{T}|{\psi }_{i}\rangle$ after *T* steps from an initial state $|{\psi }_{i}\rangle$. To characterize this nonequilibrium state, we focus on the overlap with another state $|{\psi }_{f}\rangle$, i.e., 〈*ψ*_*f*_∣*U*^*T*^∣*ψ*_*i*_〉. The logarithm of the absolute value of this overlap per system size, *F*_*L*,*T*_, is dubbed as the dynamical free energy density^11^. We here consider three types of dynamical free energy density. The first one is to take $|{\psi }_{i}\rangle =|{\psi }_{f}\rangle =|\psi \rangle$ and average the overlap over $|\psi \rangle$ randomly taken from the unitary Haar measure before taking the absolute value and the logarithm. Then, the (modified) dynamical free energy density reads

2 $${F}_{L,T}^{{{{{{{{\rm{Tr}}}}}}}}}(b,J,h)=-\frac{1}{L}{{{{{{\mathrm{log}}}}}}}\,| {{{{{{{\rm{Tr}}}}}}}}[{U}^{T}]| +{{{{{{\mathrm{log}}}}}}}\,2.$$

We note that ${F}_{L,T}^{{{{{{{{\rm{Tr}}}}}}}}}$ is the logarithm of the two-point spectral measure through $| {{{{{{{\rm{Tr}}}}}}}}[{U}^{T}]{| }^{2}={\sum }_{a,b}{{{{{{\mathrm{e}}}}}}}^{{{{{{\mathrm{i}}}}}}T({z}_{a}-{z}_{b})}$, where ${{{{{{\mathrm{e}}}}}}}^{{{{{{\mathrm{i}}}}}}{z}_{a}}$ are the eigenvalues for *U*. Since the appearance of trace simplifies the discussion, we mainly use this quantity to show our results.

The second one is to take $|{\psi }_{i}\rangle ={\bigotimes }_{j = 1}^{L}|{\uparrow }_{j}\rangle$ and $|{\psi }_{f}\rangle { = \bigotimes }_{j = 1}^{L}|{\downarrow }_{j}\rangle$, where $|{\uparrow }_{j}\rangle$/$|{\downarrow }_{j}\rangle$ is the eigenstate of ${\sigma }_{j}^{z}$ with an eigenvalue +1/−1. In this case, we have

3 $${F}_{L,T}^{\downarrow \uparrow }(b,J,h)=-\frac{1}{L}{{{{{{\mathrm{log}}}}}}}\,| \langle \downarrow \cdots \downarrow | {U}^{T}| \uparrow \cdots \uparrow \rangle | .$$

The third one is to take $|{\psi }_{i}\rangle =|{\psi }_{f}\rangle { = \bigotimes }_{j = 1}^{L}|{\uparrow }_{j}\rangle$, leading to ${F}_{L,T}^{\uparrow \uparrow }(b,J,h)=-\frac{1}{L}{{{{{{\mathrm{log}}}}}}}\,| \langle \uparrow \cdots \uparrow | {U}^{T}| \uparrow \cdots \uparrow \rangle |$.

The derivative of *F*_*L*,*T*_ gives the (imaginary part of) so-called generalized expectation values. For example, we have

4 $$-\frac{1}{T}\frac{{{{{{{{\rm{d}}}}}}}}{F}_{L,T}^{{{{{{{{\rm{Tr}}}}}}}}}}{{{{{{{{\rm{d}}}}}}}}b}={{{{{{{\rm{Im}}}}}}}}\left[{{{{{{{\rm{Tr}}}}}}}}\left(\frac{1}{L}\mathop{\sum}\limits_{j}{\sigma }_{j}^{x}\tilde{\rho }\right)\right]:= {{{{{{{\rm{Im}}}}}}}}\left[{\langle {\sigma }_{1}^{x}\rangle }_{{{{{{{{\rm{gexp}}}}}}}}}\right],$$

where $\tilde{\rho }={U}^{T}/{{{{{{{\rm{Tr}}}}}}}}[{U}^{T}]$ and we have used translation invariance. Importantly, the dynamical free energy density and the generalized expectation values can be in principle measured with an interferometric experiment^11,15^.

We seek for singularities of *F*_*∞*,*T*_ when some continuous parameter is varied. In ref. ^10^, *F*_*∞*,*T*_ exhibits singularity at critical times for continuous-time models. Since *T* is discrete in our model, instead of changing *T*, we consider continuously changing other parameters (such as *b*) for fixed *T*.

### Dynamical phases and their transitions

**Fig. 2:**
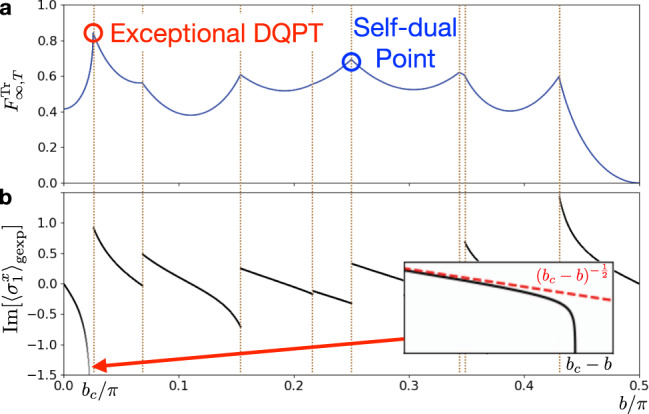
Dynamical quantum phase transitions (DQPTs) caused by the variation of the rotation angle *b*. **a** Dynamical free energy density ${F}_{\infty ,T}^{{{{{{{{\rm{Tr}}}}}}}}}$, whose singularities indicate DQPTs. Among DQPTs, we have the exceptional DQPT (red circle), which shows divergent derivative, and the DQPT crossing the self-dual point (blue circle). **b** Imaginary part of the generalized expectation value given in Eq. (4), which is proportional to the *b*-derivative of ${F}_{\infty ,T}^{{{{{{{{\rm{Tr}}}}}}}}}$. While it exhibits a jump for typical DQPTs, it diverges at the exceptional DQPT. (inset) The divergence obeys ${({b}_{c}-b)}^{-1/2}$ (red dashed line). We use *J* = −*π*/4 and *h* = 3.0, and *T* = 6.

As a prime example that highlights our discovery, we show in Fig. 2 the (real-part of) dynamical free energy density ${F}_{\infty ,T( = 6)}^{{{{{{{{\rm{Tr}}}}}}}}}$ and ${{{{{{{\rm{Im}}}}}}}}[{\langle {\sigma }_{1}^{x}\rangle }_{{{{{{{{\rm{gexp}}}}}}}}}]$ as a function of the rotation angle *b* for *J* = −*π*/4 and *h* = 3.0 (see Supplementary Note 1 for the data with other parameters and initial/final states). This is calculated from the eigenvalue with the largest modulus of the space-time dual operator, as detailed later. We find different singular behaviors for ${F}_{\infty ,T}^{{{{{{{{\rm{Tr}}}}}}}}}$, signaling distinct DQPTs at critical parameters. Many cusps of ${F}_{\infty ,T}^{{{{{{{{\rm{Tr}}}}}}}}}$ with varying *b* are are analogous to (continuous time) DQPTs studied previously, where ${{{{{{{\rm{Im}}}}}}}}[{\langle {\sigma }_{1}^{x}\rangle }_{{{{{{{{\rm{gexp}}}}}}}}}]$ exhibits a finite jump.

Notably, we find a distinct singularity at *b* = *b*_*c*_ ≃ 0.0257 for *T* = 6, where the derivative diverges as $\frac{{{{{{{{\rm{d}}}}}}}}{F}_{\infty ,T}^{{{{{{{{\rm{Tr}}}}}}}}}}{{{{{{{{\rm{d}}}}}}}}b}\propto {{{{{{{\rm{Im}}}}}}}}[{\langle {\sigma }_{1}^{x}\rangle }_{{{{{{{{\rm{gexp}}}}}}}}}] \sim | {b}_{c}-b{| }^{-1/2}$ for *b* ≲ *b*_*c*_. Such a strong singularity is prohibited for equilibrium free energy density since the thermal expectation value of a local observable cannot diverge. We call this transition an exceptional DQPT, as it turns out to originate from the occurrence of an exceptional point of a nonunitary operator that is dual to *U*. As shown below, an exceptional DQPT can occur for ${F}_{\infty ,T}^{\{{{{{{{{\rm{Tr}}}}}}}}/\downarrow \uparrow \}}$ with $J=\frac{\pi }{4}+\frac{n\pi }{2}\ (n\in {\mathbb{Z}})$ and even/odd *T* and is robust under certain weak perturbation (such as *h*), which is deeply related to the hidden symmetry of our setup. We note that the value of *b*_*c*_ itself depends on the parameters, such as *T*. We also note that, while the divergence of the derivative of dynamical free energy was recently found in ref. ^23^ for an integrable system, the connection to the underlying symmetry was not discussed.

The exceptional DQPT occurs at a different point from the self-dual points, which are $J=\frac{\pi }{4}+\frac{n\pi }{2}$ and $b=\frac{\pi }{4}+\frac{m\pi }{2}\ (n,m\in {\mathbb{Z}})$ and known in the context of quantum many-body chaos^31,32^. As discussed in Supplementary Note 5, we find that crossing self-dual points entail DQPT universally for ${F}_{\infty ,T}^{{{{{{{{\rm{Tr}}}}}}}}/\uparrow \uparrow /\downarrow \uparrow }$ with any *T* and *h*, whose criticality is analogous to that for the conventional DQPT (see Fig. 2).

We stress that DQPTs in our model do not appear as infinite-time averages of expectation values of local observables (see Supplementary Note 4), in contrast with the observation in ref. ^22^. Indeed, our DQPTs occur at nonintegrable points, where the infinite-time averages of expectation values trivially thermalize because of the Floquet eigenstate thermalization hypothesis^36^. This means that our DQPTs are unique to finite-time regimes, in which time serves as an important parameter in stark contrast with conventional phase transitions.

### Spacetime duality and hidden symmetries

To understand the above behaviors, we employ the space-time duality^30^ of our Floquet operator. This is an exact method to switch the role of time and space and rewrite *U*^*T*^ with *L* product of a space-time-dual transfer matrix $\tilde{U}$, which involves *T* spins. Using this method, we can rewrite the dynamical free energy as

5 $${F}_{L,T}=-\frac{1}{L}{{{{{{\mathrm{log}}}}}}}\,| {{{{{{{\rm{Tr}}}}}}}}[{\tilde{U}}^{L}]| ,$$

where the nonunitary operator $\tilde{U}$ depends on the type of *F*_*L*,*T*_. For example, we have

6 $${\tilde{U}}_{{{{{{{{\rm{Tr}}}}}}}}}=C{{{{{{\mathrm{e}}}}}}}^{{{{{{\mathrm{-i}}}}}}\mathop{\sum }\nolimits_{\tau = 1}^{T}\tilde{b}{\sigma }_{\tau }^{x}}{{{{{{\mathrm{e}}}}}}}^{{{{{{\mathrm{-i}}}}}}\mathop{\sum }\nolimits_{\tau = 1}^{T}\tilde{J}{\sigma }_{\tau }^{z}{\sigma }_{\tau +1}^{z}{{{{{\mathrm{-i}}}}}}\mathop{\sum }\nolimits_{\tau = 1}^{T}h{\sigma }_{\tau }^{z}}$$

with the periodic boundary condition for ${F}_{L,T}^{{{{{{{{\rm{Tr}}}}}}}}}$^30–32^ (see Supplementary Note 2 for the proof and the similar construction for ${\tilde{U}}_{\uparrow \uparrow /\downarrow \uparrow }$, which corresponds to ${F}_{L,T}^{\uparrow \uparrow /\downarrow \uparrow }$). Here, $\tilde{b}=-\pi /4-{{{{{\mathrm{i}}}}}}{{{{{{\mathrm{log}}}}}}}\,(\tan J)/2$, $\tilde{J}=-\pi /4-{{{{{\mathrm{i}}}}}}{{{{{{\mathrm{log}}}}}}}\,(\tan b)/2$ and $C={(\sin 2b/\sin 2\tilde{b})}^{T/2}/2$.

Let ${\lambda }_{{{{{{{{\rm{M}}}}}}}},\alpha }=| {\lambda }_{{{{{{{{\rm{M}}}}}}}}}| {{{{{{\mathrm{e}}}}}}}^{{{{{{\mathrm{i}}}}}}{\theta }_{\alpha }}$ be eigenvalues of $\tilde{U}$ whose modulus gives the largest one among all eigenvalues. Here, *α*(=1, … , *n*_deg_) is the label of the degeneracy, where *n*_deg_ is the number of eigenvalues giving the maximum modulus. For large *L*, *F*_*L*,*T*_ is dominated by these largest eigenvalues, i.e.,

7 $${F}_{L,T}\simeq -{{{{{{\mathrm{log}}}}}}}\,| {\lambda }_{{{{{{{{\rm{M}}}}}}}}}| -\frac{1}{L}{{{{{{\mathrm{log}}}}}}}\,\left|\mathop{\sum}\limits_{\alpha }{{{{{{\mathrm{e}}}}}}}^{{{{{{\mathrm{i}}}}}}{\theta }_{\alpha }L}\right|.$$

In the thermodynamic limit, the second term vanishes.

**Fig. 3:**
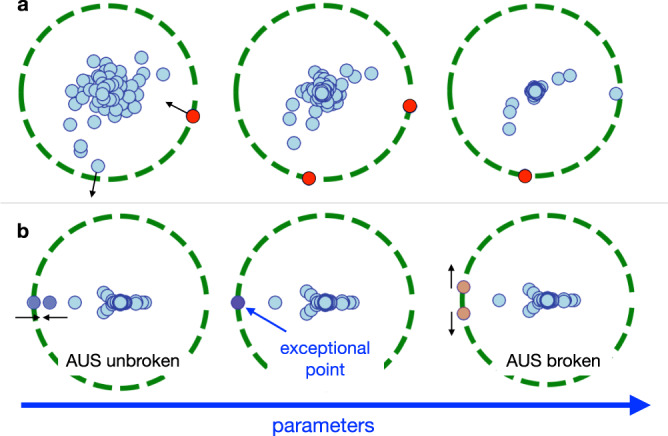
Schematic of eigenvalue dynamics of the spacetime-dual operator $\,{\tilde{\!U}}$. **a** Typical eigenvalue dynamics (small circles) near dynamical quantum phase transition (DQPT). Green dashed circles have the radius that corresponds to the eigenvalue(s) with the largest modulus. The eigenvalue with the largest modulus (red circles) switches at the critical point, at which two eigenvalues have the same modulus. **b** Eigenvalue dynamics through the exceptional DQPT. Eigenvalues with the largest and the second-largest modulus lie on the same radial direction protected by antiunitary symmetry (AUS) of ${\tilde{\!U}}$ when AUS is unbroken. When the parameter changes, the eigenvalues coincide at the critical parameter and show spectral singularity as an exceptional point. They then form a complex-conjugate pair (i.e., AUS breaking) and the modulus of two eigenvalues becomes equivalent.

Similar to the discussion noted in ref. ^14^, DQPTs occur when the eigenstate that gives the largest eigenvalue switches. For typical cases, conventional DQPTs occur when a maximum of two eigenvalues with different *θ*_*α*_ switches accidentally, where *n*_deg_ = 1 for each phase and *n*_deg_ = 2 at transition (Fig. 3a).

In contrast, hitherto unknown dynamical phases and transitions can appear when $\tilde{U}$ possesses AUS^37–39^. In nonunitary physics, the operator $\tilde{U}$ is said to have the AUS when some unitary operator *V* and $\phi \in {\mathbb{R}}$ exist and $V{\tilde{U}}^{* }{V}^{{{{\dagger}}} }={{{{{{\mathrm{e}}}}}}}^{{{{{{\mathrm{i}}}}}}\phi }\tilde{U}$ is satisfied (see Table 1). As detailed in the “Methods” section, nonunitary operator $\tilde{U}$ is called Class A if $\tilde{U}$ does not have the AUS, Class AI when the AUS exists and the corresponding *V* satisfies $V{V}^{* }={\mathbb{I}}$, and Class AII when the AUS exists and the corresponding *V* satisfies $V{V}^{* }={\mathbb{I}}$. A particularly important class is Class AI, where the spectral transition unique to nonunitarity, i.e., spontaneous AUS breaking, occurs with the change of parameters. In this case, the eigenstates do and do not respect the AUS for each phase separated at the critical point, which is called the exceptional point. Through the transition, two eigenvalues are attracted, degenerated (at the exceptional point), and repelled in a singular manner (see Fig. 3b).

**Table 1:** Summary of antiunitary symmetry (AUS) classes. Only Class AI can exhibit the AUS-breaking transition.

AUS class	$V{\tilde{U}}^{* }{V}^{{{{\dagger}}} }={{{{{{\mathrm{e}}}}}}}^{{{{{{\mathrm{i}}}}}}\phi }\tilde{U}$?	AUS-breaking transition
Class A	No	No
Class AI	$V{V}^{* }={\mathbb{I}}$	Yes
Class AII	$V{V}^{* }=-{\mathbb{I}}$	No

We find that some of our Floquet operators *U* can have hidden AUS of $\tilde{U}$ for $J=\frac{\pi }{4}+\frac{n\pi }{2}\ (n\in {\mathbb{Z}})$ (see Table 2 and “Methods” section). Particularly, ${\tilde{U}}_{{{{{{{{\rm{Tr}}}}}}}}}$ belongs to Class AI for even *T* (and AII for odd *T*), and ${\tilde{U}}_{\downarrow \uparrow }$ belongs to Class AI for odd *T* (and AII for even *T*) as long as $J=\frac{\pi }{4}+\frac{n\pi }{2}\ (n\in {\mathbb{Z}})$. In contrast, ${\tilde{U}}_{\uparrow \uparrow }$ does not have AUS and belongs to Class A in general.

**Table 2:** Antiunitary symmetry classes for $\tilde{U}$.

$\tilde{U}$ with $J=\frac{\pi }{4}+\frac{n\pi }{2}$	even *T*	odd *T*
${\tilde{U}}_{{{{{{{{\rm{Tr}}}}}}}}}$	Class AI	Class AII
${\tilde{U}}_{\downarrow \uparrow }$	Class AII	Class AI
${\tilde{U}}_{\uparrow \uparrow }$	Class A	Class A

We consider the case with $J=\frac{\pi }{4}+\frac{n\pi }{2}\ (n\in {\mathbb{Z}})$. The AUS-breaking transition can occur for Class AI, which corresponds to ${\tilde{U}}_{{{{{{{{\rm{Tr}}}}}}}}}$ with even *T* and ${\tilde{U}}_{\downarrow \uparrow }$ with odd *T*.

The above symmetries clearly explain the origin of the exceptional DQPT: as shown in Fig. 3b, this transition occurs when eigenvalues with the largest and the second-largest modulus collide under Class AI AUS, i.e., at the many-body exceptional point^40–42^ for *λ*_M_. It is known that this (second-order) exceptional point entails a universal spectral singularity, where the gap between two eigenvalues behave like ∣*b* − *b*_*c*_∣^1/2^. This leads to the previously-mentioned notable divergence of the generalized expectation value $\sim {({b}_{c}-b)}^{-1/2}$ for *b* < *b*_*c*_, where −1/2 is also known to be a universal critical exponent.

**Fig. 4:**
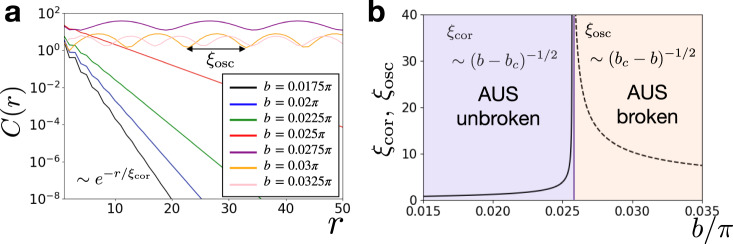
Generalized correlation function and correlation/oscillation lengths *ξ*_cor_, *ξ*_osc_ corresponding to ${F}_{\infty ,T}^{{{{{{{{\rm{Tr}}}}}}}}}$. **a** Generalized correlation function *C*(*r*) for different values of *b* for *L* = 500. In the antiunitary symmetry (AUS) unbroken phase (*b* < *b*_*c*_ ≃ 0.0257*π*), the correlation decays exponentially. In the AUS broken phase (*b* > *b*_*c*_), the correlation exhibits oscillatory long-range order. **b** Divergence of *ξ*_cor_ (solid line) and *ξ*_osc_ (dotted line) in the thermodynamic limit. Approaching the exceptional dynamical quantum phase transition, they both behave as ~∣*b* − *b*_*c*_∣^−1/2^. We use *J* = −*π*/4 and *h* = 3.0, and *T* = 6.

For ${F}_{\infty ,T = 6}^{{{{{{{{\rm{Tr}}}}}}}}}$, the phases with *b* < *b*_*c*_ ≃ 0.0257*π* and *b* > *b*_*c*_ correspond to hidden AUS-unbroken and AUS-broken phases, respectively. This is highlighted by the generalized correlation function, $C(r)=| {\langle {\sigma }_{1}^{z}{\sigma }_{r+1}^{z}\rangle }_{{{{{{{{\rm{gexp}}}}}}}}}-{\langle {\sigma }_{1}^{z}\rangle }_{{{{{{{{\rm{gexp}}}}}}}}}{\langle {\sigma }_{r+1}^{z}\rangle }_{{{{{{{{\rm{gexp}}}}}}}}}|$ (see Fig. 4 and the “Methods” section). While *C*(*r*) decays exponentially as $\sim {{{{{{\mathrm{e}}}}}}}^{-r/{\xi }_{{{{{{{{\rm{cor}}}}}}}}}}$ in the AUS-unbroken phase, the correlation length diverges as ${\xi }_{{{{{{{{\rm{cor}}}}}}}}} \sim {({b}_{c}-b)}^{-1/2}$ as it approaches the exceptional DQPT point. At AUS-broken phases, *ξ*_cor_ diverges and long-range order appears. Notably, we find that *C*(*r*) oscillates with the oscillation length *ξ*_osc_, which also diverges near the exceptional DQPT ${\xi }_{{{{{{{{\rm{osc}}}}}}}}} \sim {(b-{b}_{c})}^{-1/2}$. We remark that the qualitative signature of the transition can be captured by the existence of the long-range order even for relatively small systems, which are relevant for experiments (see Supplementary Note 6).

Here, we comment on the relation with the seminal work by Lee, Yang^43,44^ and Fisher^45^, who investigated thermodynamic phase transitions by non-Hermitian operators. While our motivation is to investigate DQPTs occurring at finite times, which is different from their motivation, there exists some mathematical analogy. In fact, the exceptional DQPT can be regarded as the realization of the edge singularity of the partition-function zeros at physical (i.e., real) parameters, as discussed in the “Methods” section.

Hidden Class AI AUS also enables us to discuss conditions for having exceptional DQPTs. In our prototypical stroboscopic Ising model, we can observe the exceptional DQPT by considering ${F}_{\infty ,T}^{{{{{{{{\rm{Tr}}}}}}}}}$ with even *T* and ${F}_{\infty ,T}^{\downarrow \uparrow }$ with odd *T* under the condition $J=\pi /4+n\pi /2\ (n\in {\mathbb{Z}})$ (see Supplementary Note 3 for the example of ${F}_{L,T}^{\downarrow \uparrow }$). Note that this transition is robust even if the value of *h* is slightly perturbed since the transition is protected by AUS. We also stress that *J* cannot be generic in our anlysis: $J=\pi /4+n\pi /2\ (n\in {\mathbb{Z}})$ is important for the exceptional DQPT because it ensures the antiunitary symmetry for $\tilde{U}$. Investigation of the exceptional DQPT for other values of *J* is a future problem.

### Signature through quasi-local observables

Next, we show that the signature of our DQPTs is accessible through the expectation values of quasi-local observables, which are more experimentally friendly than the overlap itself (in other words, the DQPT affects the behavior of the expectation values of the quasi-local observables). We also demonstrate that the exceptional DQPT is easier to measure with finite-size scaling analysis than the conventional DQPT, thanks to its strong singularity. We here explain this fact by focusing on ${F}_{L,T}^{\downarrow \uparrow }$ in Eq. (3), instead of ${F}_{L,T}^{{{{{{{{\rm{Tr}}}}}}}}}$, since its operational meaning in experimental situations is more direct. We note that ${F}_{\infty ,T}^{\downarrow \uparrow }$ shows the exceptional DQPT for *b* = *b*_*c*_ ≃ 0.446*π* with *h* = 1.3, *T* = 5 and *J* = −*π*/4, where the AUS is broken for *b* < *b*_*c*_ and unbroken for *b* > *b*_*c*_ (this is opposite to the case for ${F}_{\infty ,T}^{{{{{{{{\rm{Tr}}}}}}}}}$).

To see our argument, we introduce the following quantity

8 $${F}_{L,T}^{\downarrow \uparrow (l)}=-\frac{1}{2l}{{{{{{\mathrm{log}}}}}}}\,\langle {\psi }_{i}| {P}_{f}^{(l)}(T)| {\psi }_{i}\rangle ,$$

where ${P}_{f}^{(l)}{ = \bigotimes }_{i = 1}^{l}{|\downarrow \rangle }_{i}{\langle \downarrow |}_{i}$ and ${P}_{f}^{(l)}(T)={U}^{-T}{P}_{f}^{(l)}{U}^{T}$ is the Heisenberg representation. While ${P}_{f}^{(l = L)}{ = \bigotimes }_{i = 1}^{L}{|\downarrow \rangle }_{i}{\langle \downarrow |}_{i}=|{\psi }_{f}\rangle \langle {\psi }_{f}|$ and Eq. (8) reduces to ${F}_{L,T}^{\downarrow \uparrow }$ for *l* = *L*, ${P}_{f}^{(l)}$ becomes quasi-local when *l* = O(*L*^0^) ≪ *L*^24,25^. For the latter case, Eq. (8) is represented by the standard expectation value of the quasi-local observable, which describes the presence of consecutive spin-down domain at size *l*, at time *T*. Note that such spin domains have been measured in ion experiments using single-site imaging^8,34^.

**Fig. 5:**
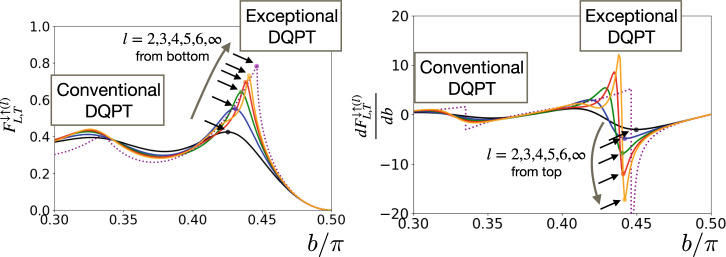
The signature of the exceptional dynamical quantum phase transition (DQPT) using quasi-local observables. We show ${F}_{L,T}^{\downarrow \uparrow (l)}$ and its derivative, which are described by the expectation value of quasi-local observables. We find that the peaks (indicated by the dots and arrows) develop even for small *l* around the exceptional DQPT (*b* ≃ 0.44*π*). Particularly, the peaks for the derivative become rapidly sharper as increasing *l*, reflecting the divergence for *l* = *L* → *∞*. This is in contrast with the conventional DQPT (*b* ≃ 0.33*π*), where we cannot find sharp peaks for *l* ≤ 6. We use *L* = 100 for *l* = 2 (black), 3 (blue), 4 (green), 5 (red), 6 (orange) and *L* = *∞* for *l* = *∞* (purple dotted), *T* = 5, *J* = −0.25*π* and *h* = 1.3.

We argue that the signature of the exceptional DQPT can be captured by ${F}_{L,T}^{\downarrow \uparrow (l)}$ and its derivative even for relatively small *l*, which is more experimentally friendly than the dynamical free energy density itself. Figure 5 shows the *b*-dependence of ${F}_{L,T}^{\downarrow \uparrow (l)}$ and $\partial {F}_{L,T}^{\downarrow \uparrow (l)}/\partial b$ for different *l*(=2, 3, 4, 5, 6, *∞*). We find that the peak develops even for small *l* around the exceptional DQPT (*b* ≃ 0.44*π*). Particularly, the peaks for the derivative become rapidly sharper as increasing *l*, reflecting the divergence for *l* = *L* → *∞*. Our results physically mean that, in this setting, large spin-down domains are rapidly suppressed toward the exceptional DQPT critical point.

We also note that the sharp peaks indicate the experimental advantage of considering the exceptional DQPT compared with the conventional DQPT. Indeed, as shown in Fig. 5, we cannot find sharp peaks for *l* ≤ 6 for the conventional DQPT (*b* ≃ 0.33*π*). This indicates that the exceptional DQPT is easier to detect even with small *l* than the conventional DQPT because of its unique singularity, which is another advantage for our analysis.

## Discussion

Although we have demonstrated the singularity of the dynamical free energy and the oscillatory long-range order for the spontaneous antiunitary symmetry breaking, one may wonder whether we can define an order parameter that is nonzero only for the symmetry-breaking phase. As detailed in Supplementary Note 7, we show that an order parameter can be explicitly constructed using different-time generalized observables. This indicates that antiunitary symmetry breaking cannot be diagnosed by the usual single-time expectation values.

The exceptional DQPT appears in other situations, as well as the above situation. When we change *h* instead of *b*, AUS of ${\tilde{U}}_{{{{{{{{\rm{Tr}}}}}}}}/\downarrow \uparrow }$ is preserved and the exceptional DQPT appears for even/odd *T*, meaning that ${\langle {\sigma }_{i}^{z}\rangle }_{{{{{{{{\rm{gexp}}}}}}}}}$ diverges. We also stress that the exceptional DQPT is not restricted to the stroboscopic Ising model but occurs for a broader class of Floquet systems, as shown in Supplementary Note 8.

To conclude, we have shown that the spontaneous antiunitary symmetry breaking leads to the unconventional universal DQPT, i.e., the exceptional DQPT, uniquely at finite times in Floquet quantum many-body systems. The appearance of finite-time phase transitions related to nonunitary physics can be understood from the spacetime duality. We have also demonstrated that the signatures of the exceptional DQPTs are observed through quasi-local observables that are accessible by state-of-the-art experiments^8,34^. Notably, the signature of the exceptional DQPTs is easier to observe than that of the normal DQPTs because of their strong singularity.

Our result paves the way to study completely unknown phases in short-time regimes, where time is regarded as a crucial parameter. As demonstrated in this work, our method via spacetime duality is useful for investigating unconventional finite-time phase transitions for quantum many-body unitary dynamics through the scope of nonunitary many-body physics. One of the promising directions is to classify such dynamical phases by the symmetries of the spacetime-dual operator in light of non-Hermitian symmetries, which are completely classified only recently^37,39^.

## Methods

### Antiunitary symmetry of $\tilde{U}$

Let us assume that a nonunitary operator $\tilde{U}$ satisfies $V{\tilde{U}}^{* }{V}^{{{{\dagger}}} }={{{{{{\mathrm{e}}}}}}}^{{{{{{\mathrm{i}}}}}}\phi }\tilde{U}$ for some unitary operator *V* and $\phi \in {\mathbb{R}}$. According to the recent classification of non-Hermitian systems^39^, $\tilde{U}$ is called Class A without AUS, Class AI when *V* with $V{V}^{* }={\mathbb{I}}$ exists, and Class AII when *V* with $V{V}^{* }=-{\mathbb{I}}$ exists. If we consider *ϕ* = 0 without loss of generality, the eigenvalues of $\tilde{U}$ in Class AI are either real or form complex conjugate pairs. Furthermore, at certain parameters, two real eigenvalues collide and form a complex conjugate pair, which can be called spontaneous AUS-breaking transition. In fact, while eigenstates $|\phi \rangle$ are symmetric under AUS in the AUS-unbroken phase, i.e., $V{|\phi \rangle }^{* }=|\phi \rangle$, $V{|\phi \rangle }^{* }$ and $|\phi \rangle$ are different in the AUS-broken phase. At the transition point, known as the exceptional point, two eigenstates become equivalent, which offers a unique feature for nonnormal matrices. In Class AII, on the other hand, eigenvalues generically form complex conjugate pairs and are not real in the presence of the level repulsion^46^.

Our Floquet operators *U* can have such hidden antiunitary symmetries of $\tilde{U}$ for $J=\frac{\pi }{4}+\frac{n\pi }{2}\ (n\in {\mathbb{Z}})$: indeed, we find $V=\mathop{\prod }\nolimits_{\tau = 1}^{T}{{{{{{\mathrm{e}}}}}}}^{{{{{{\mathrm{i}}}}}}\frac{\pi }{2}{\sigma }_{\tau }^{y}}$ for ${\tilde{U}}_{{{{{{{{\rm{Tr}}}}}}}}}$ and $V={{{{{{{\mathcal{P}}}}}}}}\mathop{\prod }\nolimits_{\tau = 1}^{T-1}{{{{{{\mathrm{e}}}}}}}^{{{{{{\mathrm{i}}}}}}\frac{\pi }{2}{\sigma }_{\tau }^{y}}$ for ${\tilde{U}}_{\downarrow \uparrow }$, where ${{{{{{{\mathcal{P}}}}}}}}$ is the parity operator exchanging *τ* and *T* − *τ* (see Supplementary Note 3 for the detailed calculation). Since VV^*^ takes either $+{\mathbb{I}}$ or $-{\mathbb{I}}$ depending on *T*, we find that ${\tilde{U}}_{{{{{{{{\rm{Tr}}}}}}}}}$ belongs to Class AI for even *T* and AII for odd *T*, and that ${\tilde{U}}_{\downarrow \uparrow }$ belongs to Class AI for odd *T* and AII for even *T* as long as $J=\frac{\pi }{4}+\frac{n\pi }{2}\ (n\in {\mathbb{Z}})$. On the other hand, ${\tilde{U}}_{\uparrow \uparrow }$ does not have AUS and belongs to Class A in general.

### Generalized correlation function

To calculate the generalized correlation function, we first note the dual representation

9 $$C(r)=\left|\frac{{{{{{{{\rm{Tr}}}}}}}}[{\tilde{U}}^{L-r}{\sigma }_{\tau = 1}^{z}{\tilde{U}}^{r}{\sigma }^{z}_{τ=1}]}{{{{{{{{\rm{Tr}}}}}}}}[{\tilde{U}}^{L}]}-{\left(\frac{{{{{{{{\rm{Tr}}}}}}}}[{\tilde{U}}^{L}{\sigma }_{\tau = 1}^{z}]}{{{{{{{{\rm{Tr}}}}}}}}[{\tilde{U}}^{L}]}\right)}^{2}\right|.$$

Here, we choose the time point *τ* for the dual spin ${\sigma }_{\tau }^{z}$ as *τ* = 1. Inserting the eigenstate decomposition of $\tilde{U}={\sum }_{\alpha }{\lambda }_{\alpha }\left|{\phi }_{\alpha }\right\rangle \left\langle {\chi }_{\alpha }\right|$, we have

10 $$C(r)\to \left|{\left(\frac{{\lambda }_{1}}{{\lambda }_{0}}\right)}^{r}\langle {\chi }_{0}| {\sigma }_{\tau = 1}^{z}| {\phi }_{1}\rangle \langle {\chi }_{1}| {\sigma }_{\tau = 1}^{z}| {\phi }_{0}\rangle \right|$$

for *b* ≲ *b*_*c*_ and large *L*. Here, 0 and 1 respectively indicate the labels of eigenvalues with the largest and the second-largest modulus. From this, the generalized correlation length is obtained as ${\xi }_{{{{{{{{\rm{cor}}}}}}}}}=-{({{{{{{\mathrm{ln}}}}}}}\,{\lambda }_{1}/{\lambda }_{0})}^{-1}\simeq {\lambda }_{0}/({\lambda }_{0}-{\lambda }_{1})$ and behaves as $\sim {({b}_{c}-b)}^{-1/2}$ near the exceptional DQPT.

For *b* > *b*_*c*_, *C*(*r*) contains a term e^−i*r*Δ^ even in the thermodynamic limit, where Δ ( < *π*) is the difference between angles of two complex-conjugate eigenvalues. Thus the oscillation length becomes ${\xi }_{{{{{{{{\rm{osc}}}}}}}}}=\frac{2\pi }{{{\Delta }}}$ and behaves as $\sim {(b-{b}_{c})}^{-1/2}$ near the exceptional DQPT.

### Partition-function zeros

Phase transitions occur when the zeros of the partition function ${{{{{{\mathrm{e}}}}}}}^{-L{F}_{L,T}}$, whose parameter (especially *b* in our context) regime is extended to a complex one, accumulate at real values in the thermodynamic limit^11,45^. Accumulation points of the partition-function zeros are thus read out from the points where maximum eigenvalues switch when we add proper perturbation $\delta b\ (\in {\mathbb{C}})$ whose magnitude is infinitesimal^14^. Notably, the partition-function zeros accumulate along the real axis when the complex-conjugate pair contributes to maximum eigenvalues with *n*_deg_ = 2 owing to AUS of $\tilde{U}$. This is because one of the eigenvalues that form the complex conjugate at $b\in {\mathbb{R}}$ becomes larger and smaller than the other for *b* + *δb* and $b-\delta b\ (\delta b\in i{\mathbb{R}})$, respectively. Moreover, we find that these zeros on the real axis (say *b* ≥ *b*_*c*_) terminate at the exceptional DQPT (*b* = *b*_*c*_). This means that the exceptional DQPT corresponds to the realization of the edge singularity of the partition-function zeros at physical parameters on the real axis.

## Supplementary information

Supplementary Information

## Data Availability

All the data that support the plots and other findings of this study are available from the corresponding author upon reasonable request.

## Code availability

All the computational codes that were used to generate the data presented in this study are available from the corresponding authors upon reasonable request.
