# Supplementary material for: Exceptional dynamical quantum phase transitions in periodically driven systems
Source: Nat Commun. 2021 Sep 1;12:5108. doi: 10.1038/s41467-021-25355-3 (PMC8410804; doi:10.1038/s41467-021-25355-3)
Supplement: Supplementary file 1 — Supplementary Information [file 41467_2021_25355_MOESM1_ESM.pdf]

Supplementary Information for "Exceptional Dynamical Quantum Phase Transitions in Periodically Driven Systems"

Ryusuke Hamazaki[1]

[1]Nonequilibrium Quantum Statistical Mechanics RIKEN Hakubi Research Team, RIKEN Cluster for Pioneering Research (CPR), RIKEN iTHEMS, Wako, Saitama 351-0198, Japan

August 2, 2021

Supplementary Note 1: Dynamical phase transitions for other parameters and initial/final states

Here, we describe in detail dynamical phases and their transitions of the stroboscopic Ising model for situations different from that presented in the main text. Supplementary Figure 1 shows examples of dynamical quantum phase transitions (DQPTs) of $F^{\mathrm{Tr}}_{\infty,\mathrm{T}}$ for $T = 7$ and $T = 8$ with $J = 0.25\pi$ with varying b. As discussed in the main text and Supplementary Note 6, n_{eff} [the number of degenerate eigenvalues with the largest modulus of $\tilde{U}$] is always equal to or larger than 2 for $T = 7$ because the system belongs to Class AII ($n_{\mathrm{eff}} \geq 4$ comes from additional symmetry $\tilde{U}$, such as the translation invariance). Therefore no exceptional DQPT occurs in this case. On the other hand, we find the exceptional DQPT for $T = 8$, indicating that the exceptional DQPT is the general mechanism that can occur in systems with Class AI-type antiunitary symmetry.

Next, Supplementary Figure 2 shows the case where we vary parameters other than b. We find that the exceptional DQPT can occur for $J = 0.25\pi$ even when h is varied for fixed appropriate b, which indicates the divergence of the generalized observable $\langle \sigma_1^z \rangle_{\mathrm{gexp}}$. On the other hand, the exceptional DQPT does not occur if J is varied since antiunitary symmetry no longer exists in $\tilde{U}$.

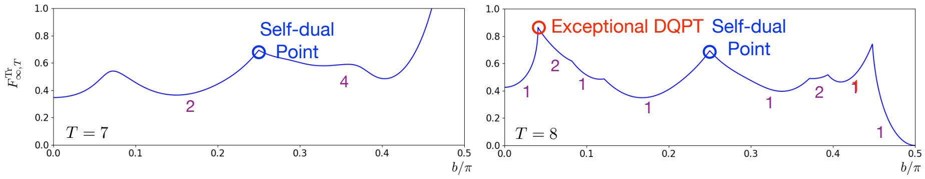

Supplementary Figure 1. **(Real part of) dynamical free energy $F^{\mathrm{Tr}}_{\infty,T}$ as a function of b for different transient times T.** As varying b, the exceptional dynamical quantum phase transition (DQPT) occurs for $T = 8$, which is prohibited for $T = 7$. Purple numbers denote n_{deg} for each phase. We use $h = 0.5$ and $J = 0.25\pi$.

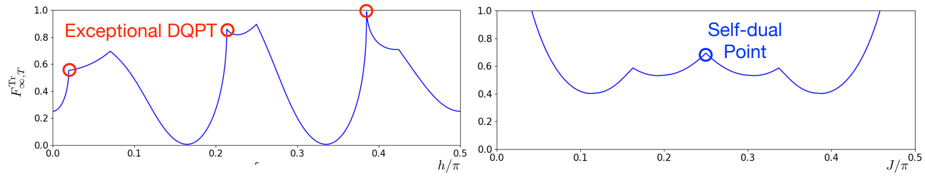

Supplementary Figure 2. **(Real part of) dynamical free energy $F^{\mathrm{Tr}}_{\infty,T}$ as a function of h and J.** As a function of h, we have several exceptional dynamical quantum phase transitions (DQPTs), where $J = 0.25\pi$, $b = 0.05\pi$ and $T = 6$ are used. On the other hand, no exceptional DQPT exists as a function of J because of the absence of the hidden antiunitary symmetry of $\tilde{U}$ ($h = 3$, $b = -0.25\pi$ and $T = 6$ are used).

Finally, Supplementary Figure 3 shows the b-dependence of $F^{\uparrow\uparrow}_{\infty,T}$ and $F^{\downarrow\uparrow}_{\infty,T}$ for $J = 0.25\pi$ and $T = 7$. As detailed in Supplementary Note 3, $\tilde{U}_{\downarrow\uparrow}$ has Class AI antiunitary symmetry for odd T, but $\tilde{U}_{\uparrow\uparrow}$ does not. Thus, while we can find the exceptional DQPT for $F^{\downarrow\uparrow}_{\infty,T}$ but not for $F^{\uparrow\uparrow}_{\infty,T}$.

Supplementary Note 2: Derivation of spacetime-dual operators

Here, we describe in detail the derivation of the spacetime-dual operators to calculate the dynamical free energies, following Refs. [1, 2, 3]. We first seek

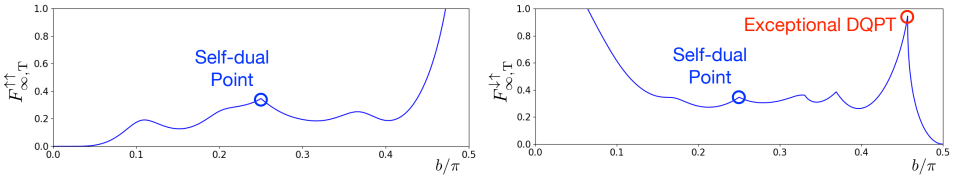

Supplementary Figure 3. **(Real part of) dynamical free energy $F^{\uparrow\uparrow}_{\infty,T}$ and $F^{\downarrow\uparrow}_{\infty,T}$ as a function of b.** The exceptional dynamical quantum phase transition (DQPT) occurs only for $F^{\downarrow\uparrow}_{\infty,T}$. Note that $n_{\rm deg}$ changes from 2 to 1 at the exceptional DQPT as increasing b. We use $h = 2, J = -0.25\pi$ and $T = 7$ for both of the data.

for the representation for $\tilde{U}_{\rm Tr}$, which satisfies

$$\frac{{\rm Tr}[U^T]}{2^L} = {\rm Tr}[\tilde{U}^L_{\rm Tr}] \tag{1}$$

or equivalently

$$F^{\rm Tr}_{L,T} = -\frac{\log|{\rm Tr}[\tilde{U}^L_{\rm Tr}]|}{L}. \tag{2}$$

For this purpose, we notice

$$\begin{aligned}{\rm Tr}[U^T] &= \sum_{\{\mathbf{s}_\tau\}}\prod_{\tau=1}^{T}\langle\mathbf{s}_{\tau+1}|e^{-i\sum_{j=1}^{L} b\sigma^x_j}e^{-i\sum_{j=1}^{L} J\sigma^z_j\sigma^z_{j+1}-i\sum_{j=1}^{L} h\sigma^z_j}|\mathbf{s}_\tau\rangle \\ &= \left(\frac{\sin 2b}{2i}\right)^{LT/2}\sum_{\{s_{\tau,j}\}} e^{-i\sum_{\tau=1}^{T}\sum_{j=1}^{L}(Js_{\tau,j}s_{\tau,j+1}+J's_{\tau,j}s_{\tau+1,j}+hs_{\tau,j})}, \end{aligned} \tag{3}$$

where $|\mathbf{s}_\tau\rangle$ are the computational basis, $s_{\tau,j}$ are classical spin variables taking ±1, and $J' = -\frac{\pi}{4} - \frac{i}{2}\log\tan b$. On the other hand, we can consider

$$\tilde{U}'_{\rm Tr} = e^{-i\sum_{\tau=1}^{T}\tilde{b}\sigma^x_\tau}e^{-i\sum_{\tau=1}^{T}(\tilde{J}\sigma^z_\tau\sigma^z_{\tau+1}+h\sigma^z_\tau)}, \tag{4}$$

which satisfies

$${\rm Tr}[\tilde{U}'^L_{\rm Tr}] = \left(\frac{\sin 2\tilde{b}}{2i}\right)^{LT/2}\sum_{\{s_{\tau,j}\}} e^{-i\sum_{\tau=1}^{T}\sum_{j=1}^{L}(\tilde{J}'s_{\tau,j}s_{\tau,j+1}+\tilde{J}s_{\tau,j}s_{\tau+1,j}+hs_{\tau,j})} \tag{5}$$

with $\tilde{J}' = -\frac{\pi}{4} - \frac{i}{2}\log\tan\tilde{b}$. Then, introducing a normalization constant

$$C = \frac{1}{2}\left(\frac{\sin 2b}{\sin 2\tilde{b}}\right)^{\frac{LT}{2}} \tag{6}$$

and setting $\tilde{b} = \arctan[e^{2i(J+\pi/4)}] = \frac{i}{2}\log\left(\frac{1+e^{2iJ}}{1-e^{2iJ}}\right) = -\frac{\pi}{4} - \frac{i}{2}\log\tan J$ (to satisfy $\tilde{J}' = J$) with $\tilde{J} = J'$, we have

$$\tilde{U}_{\mathrm{Tr}} = C\tilde{U}'_{\mathrm{Tr}} = Ce^{-i\sum_{\tau=1}^{T}\tilde{b}\sigma_\tau^x}e^{-i\sum_{\tau=1}^{T}(\tilde{J}\sigma_\tau^z\sigma_{\tau+1}^z + h\sigma_\tau^z)}, \tag{7}$$

which satisfies Supplementary Equation (1) as desired.

Next, $\tilde{U}_{\uparrow\uparrow/\downarrow\uparrow}$ can be calculated similarly. We show that they are represented as

$$\tilde{U}_{\uparrow\uparrow/\downarrow\uparrow} = C'e^{-i\sum_{\tau=1}^{T-1}\tilde{b}\sigma_\tau^x}e^{-i\sum_{\tau=1}^{T-2}\tilde{J}\sigma_\tau^z\sigma_{\tau+1}^z - i\sum_{\tau=1}^{T-1}h\sigma_\tau^z - i\tilde{J}(\sigma_1^z + I\sigma_{T-1}^z)} \tag{8}$$

with the open boundary condition, where $C' = (\sin 2b/2i)^{1/2}(\sin 2b/\sin 2\tilde{b})^{(T-1)/2}e^{-i(h+J)}$, and $I = 1(-1)$ for $\tilde{U}_{\uparrow\uparrow}(\tilde{U}_{\downarrow\uparrow})$. To see this, we notice (for $|\psi_f\rangle = |\uparrow\cdots\uparrow\rangle\,/\,|\downarrow\cdots\downarrow\rangle$)

$$\begin{aligned}\langle\psi_f|U^T|\uparrow\cdots\uparrow\rangle &= \sum_{\{\mathbf{s}_\tau\}}\langle\psi_f|U|\mathbf{s}_{T-1}\rangle\cdots\langle\mathbf{s}_1|U|\uparrow\cdots\uparrow\rangle\\ =\left(\frac{\sin 2b}{2i}\right)^{LT/2}&\sum_{\{s_{\tau,j}\}}e^{-i\sum_{\tau=1}^{T-1}\sum_{j=1}^{L}Js_{\tau,j}s_{\tau,j+1}+hs_{\tau,j} - i\sum_{\tau=1}^{T-2}\sum_{j=1}^{L}J's_{\tau,j}s_{\tau+1,j}}\\ &\times e^{-i\sum_{j=1}^{L}\{(J+h)+J's_{1,j}+J'Is_{T-1,j}\}},\end{aligned} \tag{9}$$

where $I = 1$ for $|\psi_f\rangle = |\uparrow\cdots\uparrow\rangle$ and $I = -1$ for $|\psi_f\rangle = |\downarrow\cdots\downarrow\rangle$. To construct dual operators, we consider $(T-1)$-spins along time with the open boundary condition. In fact, if we assume Supplementary Equation (8), we find

$$F_{L,T}^{\uparrow\uparrow/\downarrow\uparrow} = -\frac{\log|\mathrm{Tr}[\tilde{U}^L_{\uparrow\uparrow/\downarrow\uparrow}]|}{L}. \tag{10}$$

Supplementary Note 3: Existence of antiunitary symmetry

Here, we describe in detail the antiunitary symmetry (AUS) of the spacetime-dual operator. First, we show that the spacetime-dual operator $\tilde{U}_{\mathrm{Tr}}$ with $J = \frac{\pi}{4} + \frac{n\pi}{2}$ $(n\in\mathbb{Z})$ satisfies

$$V\tilde{U}^*_{\mathrm{Tr}}V^\dagger = e^{i\phi}\tilde{U}_{\mathrm{Tr}}, \tag{11}$$

where

$$V = \prod_{\tau=1}^{T} e^{i\frac{\pi}{2}\sigma^y_\tau} \tag{12}$$

and $\phi \in \mathbb{R}$.

For $J = \frac{\pi}{4} + \frac{n\pi}{2}$ ($n \in \mathbb{Z}$), $\tilde{b} = \pm\pi/4$ becomes real. Noticing $\tilde{J} = -\frac{\pi}{4} - \frac{i}{2}\log\tan b = -\tilde{J}^* - \frac{\pi}{2}$, the left-hand side of Supplementary Equation (11) becomes

$$\begin{aligned} V\tilde{U}^*_{\mathrm{Tr}}V^\dagger &= CVe^{i\sum_{\tau=1}^{T}\tilde{b}\sigma^x_\tau}V^\dagger V e^{i\sum_{\tau=1}^{T}(\tilde{J}^*\sigma^z_\tau\sigma^z_{\tau+1}+h\sigma^z_\tau)}V^\dagger \\ &= e^{-i\sum_{\tau=1}^{T}\tilde{b}\sigma^x_\tau}e^{i\sum_{\tau=1}^{T}(\tilde{J}^*\sigma^z_\tau\sigma^z_{\tau+1}-h\sigma^z_\tau)} \\ &= e^{-i\sum_{\tau=1}^{T}\tilde{b}\sigma^x_\tau}e^{-i\sum_{\tau=1}^{T}(\tilde{J}\sigma^z_\tau\sigma^z_{\tau+1}+h\sigma^z_\tau)}e^{-i\frac{\pi}{2}\sum_{\tau=1}^{T}\sigma^z_\tau\sigma^z_{\tau+1}} \\ &= \tilde{U}_{\mathrm{Tr}}\prod_{\tau=1}^{T}\left(-i\sigma^z_\tau\sigma^z_{\tau+1}\right) = (-i)^T\tilde{U}_{\mathrm{Tr}}, \end{aligned} \tag{13}$$

which is the right-hand side. Since $VV^* = \mathbb{I}$ for even T and $-\mathbb{I}$ for odd T, $\tilde{U}_{\mathrm{Tr}}$ belongs to Class AI for even T and Class AII for odd T.

Next, we show that $\tilde{U}_{\downarrow\uparrow}$ with $J = \frac{\pi}{4} + \frac{n\pi}{2}$ ($n \in \mathbb{Z}$) satisfies

$$V\tilde{U}^*_{\downarrow\uparrow}V^\dagger = e^{i\phi}\tilde{U}_{\downarrow\uparrow}, \tag{14}$$

where

$$V = \mathcal{P}\prod_{\tau=1}^{T-1} e^{i\frac{\pi}{2}\sigma^y_\tau} \tag{15}$$

and $\phi \in \mathbb{R}$. Here, $\mathcal{P}$ is the parity operator exchanging the site τ and $T-\tau$. In fact, the left-hand side of Supplementary Equation (14) becomes

$$\begin{aligned} V\tilde{U}^*_{\downarrow\uparrow}V^\dagger &= C'^*Ve^{i\sum_{\tau=1}^{T-1}\tilde{b}\sigma^x_\tau}V^\dagger V e^{i\sum_{\tau=1}^{T-2}\tilde{J}^*\sigma^z_\tau\sigma^z_{\tau+1}+i\sum_{\tau=1}^{T-1}h\sigma^z_\tau+i\tilde{J}^*(\sigma^z_1-\sigma^z_{T-1})}V^\dagger \\ &= e^{i\zeta}C'e^{-i\sum_{\tau=1}^{T-1}\tilde{b}\sigma^x_\tau}e^{i\sum_{\tau=1}^{T-2}\tilde{J}^*\sigma^z_\tau\sigma^z_{\tau+1}-i\sum_{\tau=1}^{T-1}h\sigma^z_\tau-i\tilde{J}^*(-\sigma^z_1+\sigma^z_{T-1})} \\ &= e^{i\zeta}\tilde{U}_{\downarrow\uparrow}e^{-i\frac{\pi}{2}\sum_{\tau=1}^{T-1}\sigma^z_\tau\sigma^z_{\tau+1}+i\frac{\pi}{2}(-\sigma^z_1+\sigma^z_{T-1})} \\ &= e^{i\zeta}\tilde{U}_{\downarrow\uparrow}\sigma^z_1\sigma^z_{T-1}\prod_{\tau=1}^{T-1}\left(-i\sigma^z_\tau\sigma^z_{\tau+1}\right) \\ &= e^{i\zeta}(-i)^{T-1}\tilde{U}_{\downarrow\uparrow}, \end{aligned} \tag{16}$$

which is the right-hand side. Here, we have used $\tilde{b} \in \mathbb{R}$ for $J = \frac{\pi}{4} + \frac{n\pi}{2} (n \in \mathbb{Z})$, $\tilde{J}^* = -(\tilde{J} + \pi/2)$, $C'^*/C' = e^{i\zeta}$ $(\zeta \in \mathbb{R})$, and $\mathcal{P}\sigma_1\mathcal{P} = \sigma_{T-1}$. Since $VV^* = \mathbb{I}$ for odd T and $-\mathbb{I}$ for even T, $\tilde{U}_{\downarrow\uparrow}$ belongs to Class AI for odd T and Class AII for even T.

We note that the minus sign associated with the exchange between $\sigma_1^z - \sigma_{T-1}^z$ under parity operation is essential for this antiunitary symmetry. This is not possible for $\tilde{U}_{\uparrow\uparrow}$, where $\sigma_1^z + \sigma_{T-1}^z$ is invariant under the parity operation.

Supplementary Note 4: Thermalization of the expectation values of local observables averaged over a long time

As mentioned in the main text, DQPTs in our model do not appear as an infinite-time average of expectation values of local observables because of the Floquet eigenstate thermalization hypothesis [4]. To demonstrate this, here we numerically show that the time-averaged expectation values of local observables become the thermalized values.

We particularly consider a time-averaged expectation value of local magnetization

$$\overline{m^z} = \frac{1}{T} \sum_{t=1}^{T} \langle \psi_i | U^{-t} \sigma_{i=1}^z U^t | \psi_i \rangle \,, \tag{17}$$

and that of local correlation

$$\overline{C^{zz}} = \frac{1}{T} \sum_{t=1}^{T} \langle \psi_i | U^{-t} \sigma_{i=1}^z \sigma_{i=2}^z U^t | \psi_i \rangle \,, \tag{18}$$

where T is sufficiently large. If the system thermalizes, $\overline{m^z}$ and $\overline{C^{zz}}$ will be equal to the expectation value at the infinite temperature, i.e., $\mathrm{Tr}[\sigma_{i=1}^z]/2^L = \mathrm{Tr}[\sigma_{i=1}^z \sigma_{i=2}^z]/2^L = 0$. In the following, we take an initial state as $|\psi_i\rangle = |\uparrow\uparrow \cdots \uparrow\rangle$. We remind that the DQPTs (including the exceptional DQPT) occur for several b for $F_{\infty,T}^{\{\uparrow\uparrow/\downarrow\uparrow\}}$ with finite T starting from this initial state (see Supplementary Note 1).

Supplementary Figure 4 shows the values of $\overline{m^z}$ and $\overline{C^{zz}}$ as a function of b for different system sizes L. We find that $\overline{m^z}$ and $\overline{C^{zz}}$ approach zero as increasing the system size especially for b far from the integrable point

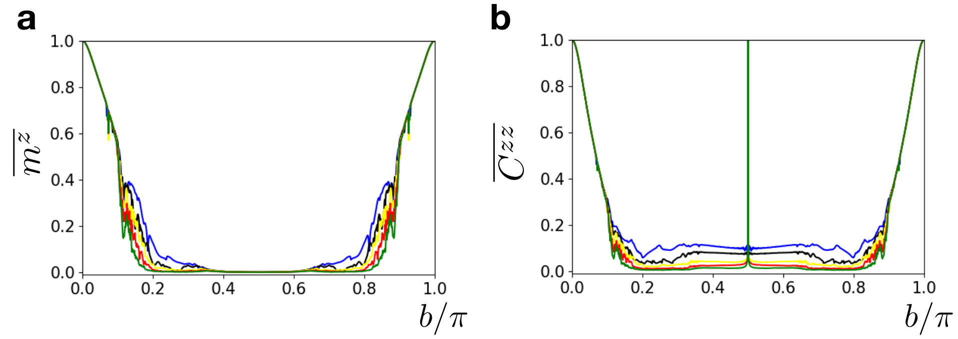

Supplementary Figure 4. **Long time average of the expectation values of local observables in a. Supplementary Equation** (17) **and b. Supplementary Equation** (18) **as a function of** b**.** We show results for different system sizes, $L = 8$ (blue), 10 (black) 12 (yellow), 14 (red), and 16 (green). We find that $\overline{m^z}$ and $\overline{C^{zz}}$ approach zero as increasing the system size especially for b far from the integrable point ($b/\pi = 0, 0.5, 1$), which indicates that they become zero in the thermodynamic limit. This means that, while we have several dynamical quantum phase transitions of $F_{\infty,T}^{\{\uparrow\uparrow/\downarrow\uparrow\}}$ with finite T for the displayed range of b, long-time-averages of the expectation values thermalize to the values described by the infinite-temperature state. We use $T = 1000$, $J = -0,25\pi$ and $h = 1.3\pi$.

($b/\pi = 0, 0.5, 1$), which indicates that they become zero in the thermodynamic limit. This implies that our DQPTs are unique to finite-time regimes, in which time serves as an important parameter in stark contrast with conventional phase transitions.

Supplementary Note 5: Dynamical phase transition at the self-dual point

In this section, we discuss the details for the DQPT occurring at the self-dual points, i.e., $J = \frac{\pi}{4} + \frac{n\pi}{2}$ and $b = \frac{\pi}{4} + \frac{m\pi}{2}$ $(n, m \in \mathbb{Z})$ [2, 3]. We especially show that crossing self-dual points entails DQPTs universally for $F_{\infty,T}^{\mathrm{Tr}/\uparrow\uparrow/\downarrow\uparrow}$ with any T and h, whose singularity is analogous to that for the conventional DQPT. Moreover, the dynamical free energy density takes a universal value as $F_{\infty,T}^{\mathrm{Tr}} = \log 2$ or $F_{\infty,T}^{\uparrow\uparrow/\downarrow\uparrow} = \log 2/2$ there.

We especially focus on the case for F_{Tr} (other dynamical free energies

are discussed in a similar manner). As discussed in the main text, we can exactly write

$$\tilde{U}_{\mathrm{Tr}} = Ce^{-i\sum_{\tau=1}^{T}\tilde{b}\sigma_\tau^x}e^{-i\sum_{\tau=1}^{T}\tilde{J}\sigma_\tau^z\sigma_{\tau+1}^z-i\sum_{\tau=1}^{T}h\sigma_\tau^z} \tag{19}$$

using the spacetime duality [1, 2, 3]. Here, $\tilde{b} = -\pi/4 - i\log(\tan J)/2$, $\tilde{J} = -\pi/4 - i\log(\tan b)/2$ and $C = (\sin 2b/\sin 2\tilde{b})^{T/2}/2$. Importantly, $\tilde{U}$ is unitary (up to a constant) only at the self-dual points.

As noted in the main text, for typical cases, DQPTs occur when maximum of two eigenvalues with different θ_α switches accidentally, where $n_{\mathrm{deg}} = 1$ for each phase and $n_{\mathrm{deg}} = 2$ at transition (Supplementary Figure 5**a**). On the other hand, DQPT occurs more universally if the self-dual point is crossed: all of the modulus of the eigenvalues of $\tilde{U}$ are equal at this point ($n_{\mathrm{deg}} = 2^T$), and crossing this point typically switches the largest eigenvalue (Supplementary Figure 5**b**). At transition, dynamical free energies are determined only by the modulus of the eigenvalues, which leads to universal values $F_{\infty,T}^{\mathrm{Tr}} = \log 2$ (similarly, we have $F_{\infty,T}^{\uparrow\uparrow/\downarrow\uparrow} = \log 2/2$). To our best knowledge, this is the first evidence that the self-dual point is a critical point of different dynamical phases.

Supplementary Note 6: Dynamical phase transitions in finite systems

Here, we show several results concerning the DQPTs for finite system size L.

Degeneracy and finite-size effect

While the number of eigenvalues with maximum modulus n_{deg} does not contribute to the free energy density for $L \to \infty$, n_{deg} can characterize each phase via a finite-size correction $\Delta F_{L,T} = F_{L,T} - F_{\infty,T}$. Indeed, the second term in

$$F_{L,T} \simeq -\log|\lambda_{\mathrm{M}}| - \frac{1}{L}\log\left|\sum_\alpha e^{i\theta_\alpha L}\right|. \tag{20}$$

shows that $\Delta F_{L,T}$ is upper bounded by $\log n_{\mathrm{deg}}/L$, where the bound is achieved when $e^{i\theta_\alpha L} = e^{i\theta_\beta L}$ for every $\alpha \neq \beta$.

Supplementary Figure 6**a** shows the finite-size scaling of $\Delta F_{L,T}^{\mathrm{Tr}}$ near the exceptional DQPT. While $\Delta F_{L,T}^{\mathrm{Tr}}$ exponentially decays with L for $b < b_c$

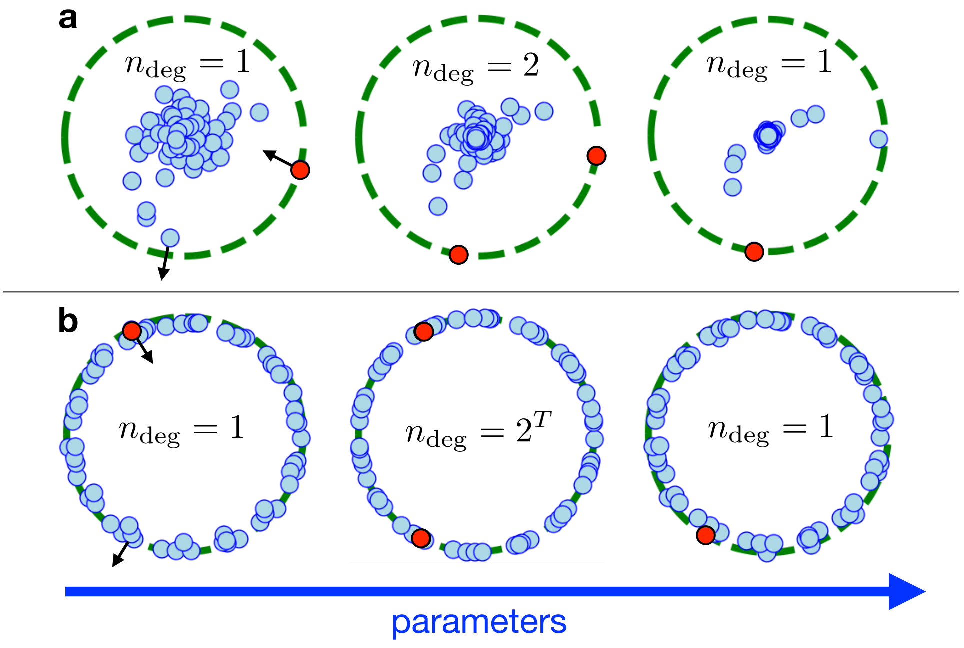

Supplementary Figure 5. **Schematic of eigenvalue dynamics of the spacetime-dual operator $\tilde{U}$ and its relation to the dynamical quantum phase transition (DQPT). a.** Typical eigenvalue dynamics (small circles) near DQPT. Green dashed circles have the radius that corresponds to the eigenvalue(s) with the largest modulus. The eigenvalue with the largest modulus (red circles) switches at the critical point, at which two eigenvalues have the same modulus $n_{\rm deg} = 2$. **b.** Eigenvalue dynamics through the DQPT induced by self-dual points. At the self-dual point, all of 2^T numbers of eigenvalues have the same modulus owing to the unitarity of $\tilde{U}$.

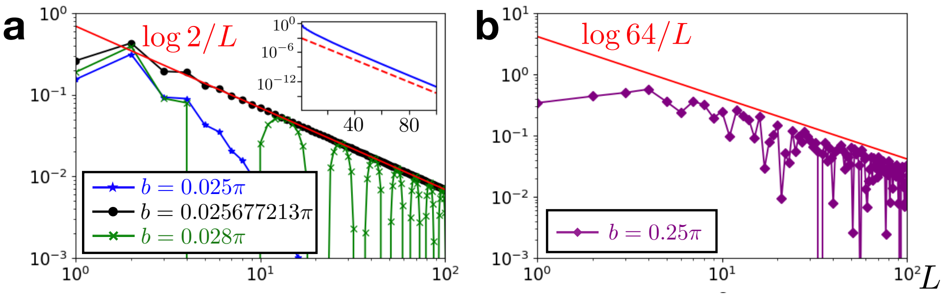

Supplementary Figure 6. **Log-log plot for finite-size correction of the dynamical free energy density $\Delta F^{\mathrm{Tr}}_{L,T}$. a.** Behavior near the exceptional dynamical quantum phase transition (DQPT). Below the DQPT with $n_{\mathrm{deg}} = 1$ (blue), $\Delta F^{\mathrm{Tr}}_{L,T}$ decays rapidly. (inset) Semi-log plot of the same data shows that it indeed decays exponentially (dotted red line is an eyeguide for exponential decay). Above the DQPT with $n_{\mathrm{deg}} = 2$ (green), $\Delta F^{\mathrm{Tr}}_{L,T}$ decays with oscillations. The decay is bounded by $\log 2/L$ (red solid line) for sufficiently large L. Approaching the critical point (black), the oscillation vanishes and $\Delta F^{\mathrm{Tr}}_{L,T} \sim \log 2/L$. **b.** Behavior at the self-dual point (purple), where $n_{\mathrm{deg}} = 2^T = 64$. The correction is bounded by $\log 64/L$. We use $J = -\pi/4$ and $h = 3.0$, and $T = 6$.

since $n_{\mathrm{deg}} = 1$, it exhibits polynomially decaying oscillations for $b > b_c$, where the decay is upper bounded by $\log n_{\mathrm{deg}}/L = \log 2/L$ for sufficiently large L. At the transition point, oscillation-free polynomial decay is observed. Another example is the behavior at the self-dual point, where the decay is upper bounded by $\log 2^T/L$ (Supplementary Figure 6**b**).

The number n_{deg} thus provides universal information through $\Delta F_{L,T}$ on dynamical phases, which is deeply related to the symmetries hidden in the space-time dual operator $\tilde{U}$. If $\tilde{U}$ is in Class AI ($F^{\mathrm{Tr}}_{L,T}$ with even T and $F^{\downarrow\uparrow}_{L,T}$ with odd T under the condition $J = -\pi/4 + n\pi/2$ ($n \in \mathbb{Z}$)), phases with $n_{\mathrm{deg}} = 2$ can naturally appear as well as phases with $n_{\mathrm{deg}} = 1$. If $\tilde{U}$ is in Class AII ($F^{\mathrm{Tr}}_{L,T}$ with odd T and $F^{\downarrow\uparrow}_{L,T}$ with even T under the condition $J = -\pi/4 + n\pi/2$ ($n \in \mathbb{Z}$)), all phases satisfy $n_{\mathrm{deg}} \geq 2$. We note that other symmetries are found to exist that enhance the value of n_{deg}, such as translation invariance for $\tilde{U}_{\mathrm{Tr}}$ or integrability at $h = 0$, which may also be interesting to study systematically.

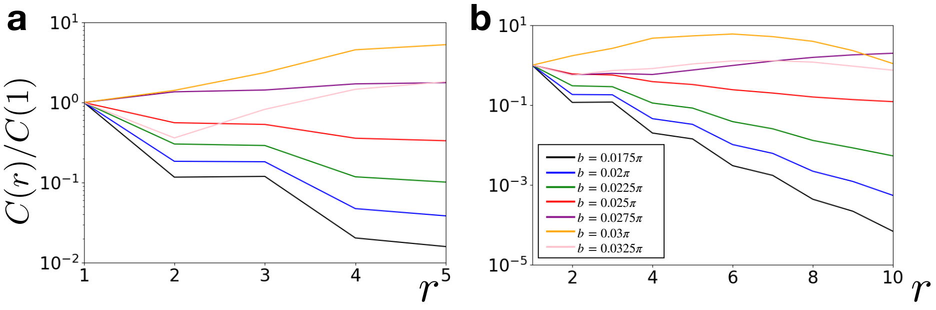

Supplementary Figure 7. **Normalized generalized correlation function $C(r)/C(0)$ for different values of b. a.** Results for $L = 10$. Clear difference between antiunitary-symmetry (AUS) unbroken ($b < b_c \simeq 0.0257\pi$) and AUS broken ($b > b_c$) regimes already appears. Indeed, $C(r)$ decays fast for the former but does not decay for the latter. **b.** Results for $L = 20$. The oscillatory behavior in the AUS-broken regime arises, where the oscillation length is evaluated as $\xi_{\rm osc} \simeq 10.4$ for $b = 0.03\pi$ and $\xi_{\rm osc} \simeq 8.5$ for $b = 0.0325\pi$. We use $J = -\pi/4$ and $h = 3.0$, and $T = 6$.

Generalized correlation function in small systems

While the true DQPT occurs in the thermodynamic limit, qualitative signature of AUS-unbroken and AUS-broken phases are already captured even for finite system sizes with the generalized correlation function. In Supplementary Figure 7, we show the (normalized) generalized correlation function for different system size L. Even for $L = 10$, which has been prepared in experiments of trapped ions [5], we find clear difference between AUS-unbroken ($b < b_c \simeq 0.0257\pi$) and AUS-broken ($b > b_c$) regime. Indeed, $C(r)$ decays fast for the former but does not decay for the latter. If we consider $L = 20$, we can also see the oscillatory behavior in the AUS-broken regime: note that the oscillation length is evaluated as $\xi_{\rm osc} \simeq 10.4$ for $b = 0.03\pi$ and $\xi_{\rm osc} \simeq 8.5$ for $b = 0.0325\pi$.

Supplementary Note 7: Explicit construction of an order parameter

We here show that we can explicitly construct an order parameter using different-time generalized observables. For this purpose, we especially focus

on $F^{\downarrow\uparrow}_{L,T} = -\frac{1}{L}\log|\langle\psi_f|U^T|\psi_i\rangle|$ with $|\psi_i\rangle = |\uparrow\cdots\uparrow\rangle$ and $|\psi_f\rangle = |\downarrow\cdots\downarrow\rangle$, instead of $F^{\mathrm{Tr}}_{L,T}$, since its operational meaning in experimental situations is more direct. We note that $F^{\downarrow\uparrow}_{\infty,T}$ shows the exceptional DQPT for $b = b_c \simeq 0.446\pi$ with $h = 1.3$, $T = 5$ and $J = -\pi/4$, where the AUS is broken for $b < b_c$ and unbroken for $b > b_c$ (note that this is opposite to the case for $F^{\mathrm{Tr}}_{\infty,T}$).

For our discussion, we first note that, for a usual symmetry breaking, such as $\mathbb{Z}_2$ symmetry breaking of an Ising model, magnetization m^z becomes an order parameter. In this case, m^z is odd under symmetry operation ($m^z \to -m^z$), so $\langle m^z\rangle = -\langle m^z\rangle = 0$ when symmetry of the state is unbroken.

Similarly, our antiunitary symmetry operation in the space-time dual space ($V = \mathcal{P}\prod_{\tau=1}^{T-1} e^{i\frac{\pi}{2}\sigma^y_\tau}$ for $\tilde{U}_{\downarrow\uparrow}$) is found to correspond to a combined symmetry of time exchange $\tau \leftrightarrow T-\tau$, complex conjugation, and certain spin reversal in the original space. For example, using the spacetime-dual transformation and the spectral decomposition $\tilde{U}_{\downarrow\uparrow} = \sum_\alpha \lambda_\alpha |\phi_\alpha\rangle\langle\chi_\alpha|$, we have

$$\langle\psi_f|U^{T-\tau}\sigma^z_i U^\tau|\psi_i\rangle = \mathrm{Tr}[\tilde{U}^L_{\downarrow\uparrow}\sigma^z_\tau] \tag{21}$$

$$\to \lambda_0^L \langle\chi_0|\sigma^z_\tau|\phi_0\rangle \tag{22}$$

for the symmetry-unbroken phase, where 0 labels the eigenvalues with the largest modulus. In this phase, we have

$$V|\phi_0\rangle^* = |\phi_0\rangle, \quad V|\chi_0\rangle^* = |\chi_0\rangle, \tag{23}$$

and thus

$$\langle\chi_0|\sigma^z_\tau|\phi_0\rangle = \langle\chi_0^*|V^\dagger\sigma^z_\tau V|\phi_0^*\rangle = -\langle\chi_0|\sigma^z_{T-\tau}|\phi_0\rangle^* \tag{24}$$

Then, we find that $\mathrm{Re}[\langle\psi_f|U^{T-\tau}\sigma^z_i U^\tau|\psi_i\rangle] = -\mathrm{Re}[\langle\psi_f|U^\tau\sigma^z_i U^{T-\tau}|\psi_i\rangle]$ if the symmetry is unbroken [the spin indices i is arbitrary]. On the other hand, we can also find $\mathrm{Re}[\langle\psi_f|U^{T-\tau}\sigma^z_i U^\tau|\psi_i\rangle] \neq -\mathrm{Re}[\langle\psi_f|U^\tau\sigma^z_i U^{T-\tau}|\psi_i\rangle]$ for the symmetry-breaking phase.

Then, we can explicitly construct the following order parameter using generalized observables:

$$M = \left|\mathrm{Re}\left[\sum_{\tau=1}^{T-1}\frac{\langle\psi_f|U^{T-\tau}\sigma^z_i U^\tau|\psi_i\rangle}{\langle\psi_f|U^T|\psi_i\rangle}\right]\right|^2. \tag{25}$$

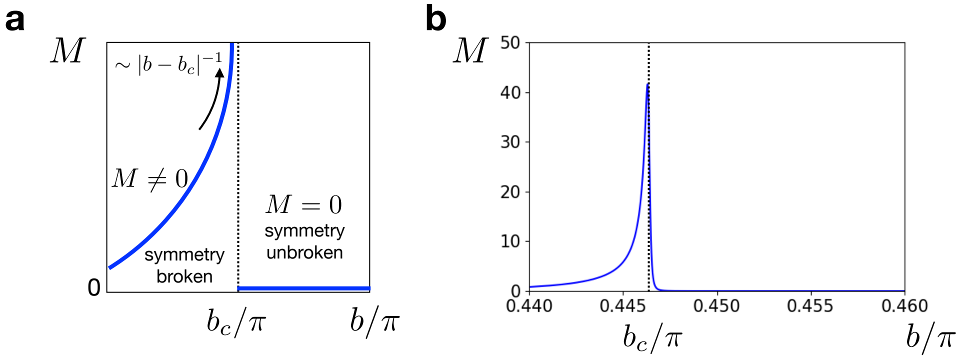

Supplementary Figure 8. **The order parameter in Supplementary Equation** (25)**.** **a,** Schematic illustration of the behavior for M for infinite system size. **b,** Numerical verification. The exceptional dynamical quantum phase transition for $F^{\downarrow\uparrow}_{L,T}$ occurs for $b = b_c \simeq 0.446\pi$, where $b < b_c$ represents the symmetry-broken and $b > b_c$ represents the symmetry-unbroken phase. We find $M \neq 0$ for $b < b_c$ and $M \simeq 0$ for $b > b_c$ (the deviation from zero and the finite peak are due to the finite symmetry-breaking term, which is required for finite L). We use $L = 800$, $T = 5$, $J = -0.2498\pi$ and $h = 1.3$.

When the antiunitary symmetry is unbroken, $M = 0$, and when it is broken, $M \neq 0$ and behaves as $\sim |b - b_c|^{-1}$ at criticality (Supplementary Figure 8**a**). As shown in Supplementary Figure 8**b**, we can demonstrate this using a numerical simulation. Here, the slight deviation from $M = 0$ for the symmetry-unbroken phase and the finite peak at the critical point are due to the finite symmetry-breaking term, which is required for finite L to demonstrate the symmetry breaking.

We note that M is constructed using the different-time generalized observables rather than a single-time expectation value of local observables. In the above example, M diagnoses symmetry of interference structure in quantum dynamics related to the time-reversal operation $\tau \leftrightarrow T - \tau$.

Supplementary Note 8: Other models that exhibit exceptional DQPT

Floquet unitary circuits

Here, we describe in detail that exceptional DQPT can occur in certain Floquet unitary circuits, in addition to our stroboscopic Ising model. We assume that the system size L is even and consider the unitary circuit given in the form as

$$U = \prod_{j:\mathrm{even}} \mathcal{U}_{j,j+1} \prod_{j:\mathrm{odd}} \mathcal{U}_{j,j+1}, \tag{26}$$

which is composed of two-site unitary circuits $\mathcal{U}_{j,j+1}$ (Supplementary Figure 9). When the dimension of the local Hilbert space is two (i.e., spin-1/2 systems), such two-site unitary circuits can be generally represented as [6]

$$\mathcal{U}_{j,j+1} = e^{i\xi}(u_j \otimes u_{j+1})\mathcal{V}(v_j \otimes v_{j+1}), \tag{27}$$

where u_j and v_j are one-site unitary operators, $\xi \in \mathbb{R}$, and $\mathcal{V}$ can be parametrized as

$$\mathcal{V} = e^{-i(J_1\sigma_j^x\sigma_{j+1}^x + J_2\sigma_j^y\sigma_{j+1}^y + J_3\sigma_j^z\sigma_{j+1}^z)} \tag{28}$$

using $J_1, J_2, J_3 \in \mathbb{R}$. For simplicity, we assume that $u_j = u$ and $v_j = v$ are j-independent in the following.

We now focus on time evolution for $T/2$ steps, which correspond to the total time $T\,(\in 2\mathbb{N})$, and the following (real part of) dynamical free energy:

$$F^{\mathrm{Tr}}_{L,T/2} = -\frac{1}{L}\log|\mathrm{Tr}[U^{T/2}]| + \log 2. \tag{29}$$

We require that the spacetime-dual operator $\tilde{U}$ of U should satisfy

$$F^{\mathrm{Tr}}_{L,T/2} = -\frac{1}{L}\log|\mathrm{Tr}[\tilde{U}^{L/2}]|, \tag{30}$$

with which $F^{\mathrm{Tr}}_{\infty,T/2}$ is given by $-(\log|\lambda_{\mathrm{M}}|)/2$.

By considering the dual operators for $\mathcal{U}_{j,j+1}$ [6], we find

$$\tilde{U} = \frac{1}{4}\prod_{\tau:\mathrm{even}} \tilde{\mathcal{U}}_{\tau,\tau+1} \prod_{\tau:\mathrm{odd}} \tilde{\mathcal{U}}_{\tau,\tau+1}, \tag{31}$$

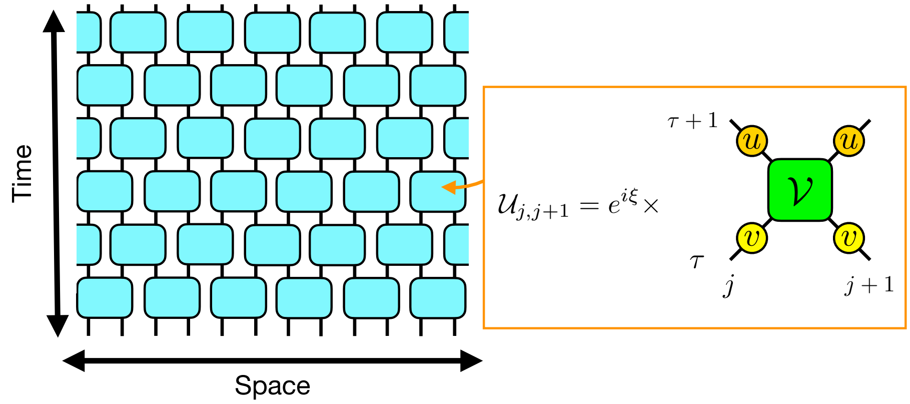

Supplementary Figure 9. **Schematic illustration of the Floquet circuit dynamics in Supplementary Equation** (26)**.** We show the example for $L = 12$ and $T = 6$. Each of the block $\mathcal{U}_{j,j+1}$ can be written as in Supplementary Equation (27).

where

$$\tilde{\mathcal{U}}_{\tau,\tau+1} = e^{i\xi}(v^{\mathrm{T}} \otimes u)\tilde{\mathcal{V}}_{\tau,\tau+1}(v \otimes u^{\mathrm{T}}). \tag{32}$$

Here,

$$\tilde{\mathcal{V}}_{\tau,\tau+1} = \frac{1}{2}e^{-iJ_3+iJ_-}\sigma^z_\tau\sigma^z_{\tau+1} + \frac{1}{2}e^{-iJ_3-iJ_-} + \frac{1}{2}e^{iJ_3-iJ_+}\sigma^x_\tau\sigma^x_{\tau+1} - \frac{1}{2}e^{iJ_3+iJ_+}\sigma^y_\tau\sigma^y_{\tau+1} \tag{33}$$

with $J_\pm = J_1 \pm J_2$ and v^{T} denotes the transposition of v. It can also be written as [6]

$$\begin{bmatrix} e^{-iJ_3}\cos(J_-) & 0 & 0 & e^{iJ_3}\cos(J_+) \\ 0 & -ie^{-iJ_3}\sin(J_-) & -ie^{iJ_3}\sin(J_+) & 0 \\ 0 & -ie^{iJ_3}\sin(J_+) & -ie^{-iJ_3}\sin(J_-) & 0 \\ e^{iJ_3}\cos(J_+) & 0 & 0 & e^{-iJ_3}\cos(J_-) \end{bmatrix}. \tag{34}$$

Antiunitary symmetry

To discuss the existence of the exceptional DQPT, we restrict ourselves to $\xi = 0$ and

$$u = v = e^{-i\frac{h}{2}\sigma^z}, \tag{35}$$

which correspond to the presence of the uniform magnetic field. To simplify the notation, we consider the unitary transformation for $\tilde{U} \to (u \otimes u)\tilde{U}(u^\dagger \otimes u^\dagger)$, which does not change its eigenvalues, and discuss

$$\tilde{U} = \frac{1}{4} \prod_{\tau:\text{even}} e^{-ih\sigma^z_\tau} e^{-ih\sigma^z_{\tau+1}} \tilde{\mathcal{V}}_{\tau,\tau+1} \prod_{\tau:\text{odd}} e^{-ih\sigma^z_\tau} e^{-ih\sigma^z_{\tau+1}} \tilde{\mathcal{V}}_{\tau,\tau+1} \tag{36}$$

In the following, we show that $\tilde{U}$ has the AUS and that the exceptional DQPT can exist for nontrivial points $J_3 = \pi/4$ and $J_3 = \pi/2$.

Let us first consider the case for $J_3 = \pi/2$. In this case, we show that

$$V = \prod_{\tau=1}^{T} e^{i\frac{\pi}{2}\sigma^x_\tau} = i^T \prod_{\tau=1}^{T} \sigma^x_\tau \tag{37}$$

becomes the AUS. To see this, we note that

$$V\tilde{U}V^\dagger = \frac{1}{4} \prod_{\tau:\text{even}} V_2 e^{-ih(\sigma^z_\tau+\sigma^z_{\tau+1})} \tilde{\mathcal{V}}_{\tau,\tau+1} V_2^\dagger \prod_{\tau:\text{odd}} V_2 e^{-ih(\sigma^z_\tau+\sigma^z_{\tau+1})} \tilde{\mathcal{V}}_{\tau,\tau+1} V_2^\dagger, \tag{38}$$

where V_2 is a shorthand notation for $\sigma^x_\tau \sigma^x_{\tau+1}$. First, nonzero matrix elements of local gates satisfy

$$\begin{aligned} \langle\uparrow\downarrow| V_2 e^{-ih(\sigma^z_\tau+\sigma^z_{\tau+1})} \tilde{\mathcal{V}}_{\tau,\tau+1} V_2^\dagger |\uparrow\downarrow\rangle &= \langle\downarrow\uparrow| e^{-ih(\sigma^z_\tau+\sigma^z_{\tau+1})} \tilde{\mathcal{V}}_{\tau,\tau+1} |\downarrow\uparrow\rangle \\ &= \langle\uparrow\downarrow| e^{-ih(\sigma^z_\tau+\sigma^z_{\tau+1})} \tilde{\mathcal{V}}_{\tau,\tau+1} |\uparrow\downarrow\rangle^* = -\sin J_- \end{aligned} \tag{39}$$

$$\begin{aligned} \langle\downarrow\uparrow| V_2 e^{-ih(\sigma^z_\tau+\sigma^z_{\tau+1})} \tilde{\mathcal{V}}_{\tau,\tau+1} V_2^\dagger |\uparrow\downarrow\rangle &= \langle\uparrow\downarrow| e^{-ih(\sigma^z_\tau+\sigma^z_{\tau+1})} \tilde{\mathcal{V}}_{\tau,\tau+1} |\downarrow\uparrow\rangle \\ &= \langle\downarrow\uparrow| e^{-ih(\sigma^z_\tau+\sigma^z_{\tau+1})} \tilde{\mathcal{V}}_{\tau,\tau+1} |\uparrow\downarrow\rangle^* = \sin J_+ \end{aligned} \tag{40}$$

$$\begin{aligned} \langle\downarrow\downarrow| V_2 e^{-ih(\sigma^z_\tau+\sigma^z_{\tau+1})} \tilde{\mathcal{V}}_{\tau,\tau+1} V_2^\dagger |\uparrow\uparrow\rangle &= \langle\uparrow\uparrow| e^{-ih(\sigma^z_\tau+\sigma^z_{\tau+1})} \tilde{\mathcal{V}}_{\tau,\tau+1} |\downarrow\downarrow\rangle \\ &= -\langle\downarrow\downarrow| e^{-ih(\sigma^z_\tau+\sigma^z_{\tau+1})} \tilde{\mathcal{V}}_{\tau,\tau+1} |\uparrow\uparrow\rangle^* = i\cos J_+ \end{aligned} \tag{41}$$

$$\begin{aligned} \langle\downarrow\downarrow| V_2 e^{-ih(\sigma^z_\tau+\sigma^z_{\tau+1})} \tilde{\mathcal{V}}_{\tau,\tau+1} V_2^\dagger |\downarrow\downarrow\rangle &= \langle\uparrow\uparrow| e^{-ih(\sigma^z_\tau+\sigma^z_{\tau+1})} \tilde{\mathcal{V}}_{\tau,\tau+1} |\uparrow\uparrow\rangle \\ &= -\langle\downarrow\downarrow| e^{-ih(\sigma^z_\tau+\sigma^z_{\tau+1})} \tilde{\mathcal{V}}_{\tau,\tau+1} |\downarrow\downarrow\rangle^* = -ie^{-2ih}\cos J_- \end{aligned} \tag{42}$$

$$\begin{aligned} \langle\uparrow\uparrow| V_2 e^{-ih(\sigma^z_\tau+\sigma^z_{\tau+1})} \tilde{\mathcal{V}}_{\tau,\tau+1} V_2^\dagger |\uparrow\uparrow\rangle &= \langle\downarrow\downarrow| e^{-ih(\sigma^z_\tau+\sigma^z_{\tau+1})} \tilde{\mathcal{V}}_{\tau,\tau+1} |\downarrow\downarrow\rangle \\ &= -\langle\uparrow\uparrow| e^{-ih(\sigma^z_\tau+\sigma^z_{\tau+1})} \tilde{\mathcal{V}}_{\tau,\tau+1} |\uparrow\uparrow\rangle^* = -ie^{2ih}\cos J_- \end{aligned} \tag{43}$$

and the other matrix elements are zero, where we have used Supplementary Equation (34) with $J_3 = \pi/2$. From this, while matrix elements of the two-site transitions for $\{|\uparrow\downarrow\rangle \to |\uparrow\downarrow\rangle, |\uparrow\downarrow\rangle \to |\downarrow\uparrow\rangle, |\downarrow\uparrow\rangle \to |\downarrow\uparrow\rangle, |\downarrow\uparrow\rangle \to |\uparrow\downarrow\rangle\}$ (yellow gates in Supplementary Figure 10) are invariant under complex conjugation, those of the two-site transitions for $\{|\uparrow\uparrow\rangle \to |\uparrow\uparrow\rangle, |\uparrow\uparrow\rangle \to |\downarrow\downarrow\rangle, |\downarrow\downarrow\rangle \to |\uparrow\uparrow\rangle, |\downarrow\downarrow\rangle \to |\downarrow\downarrow\rangle\}$ (green gates in Supplementary Figure 10) acquire a minus sign under complex conjugation.

Now, consider matrix elements of $\tilde{U}$ as a sum of the paths of the computational states. For example, we can consider a matrix element

$$\langle\uparrow\uparrow\downarrow\downarrow\downarrow\uparrow|\tilde{U}|\uparrow\downarrow\uparrow\uparrow\downarrow\downarrow\rangle = \frac{1}{4}\sum_{\mathbf{s}}\langle\uparrow\uparrow\downarrow\downarrow\downarrow\uparrow|\prod_{\tau:\mathrm{even}} e^{-ih(\sigma^z_\tau+\sigma^z_{\tau+1})}\tilde{\mathcal{V}}_{\tau,\tau+1}|\mathbf{s}\rangle \times \langle\mathbf{s}|\prod_{\tau:\mathrm{odd}} e^{-ih(\sigma^z_\tau+\sigma^z_{\tau+1})}\tilde{\mathcal{V}}_{\tau,\tau+1}|\uparrow\downarrow\uparrow\uparrow\downarrow\downarrow\rangle \quad (44)$$

for $T = 6$. In Supplementary Figure 10, we show one of the paths that corresponds to $|\mathbf{s}\rangle = |\uparrow\downarrow\uparrow\uparrow\uparrow\uparrow\rangle$ for this example. Then, generally, we can show that each of the paths must include even times of two-site transitions for green gates, and even times for yellow gates. To see this, we focus on the difference of magnetization δm between even and odd sites (in the time direction). For the above example, δm is -1 for $|\uparrow\downarrow\uparrow\uparrow\downarrow\downarrow\rangle$ and $|\uparrow\downarrow\uparrow\uparrow\uparrow\uparrow\rangle$, and $+1$ for $|\uparrow\uparrow\downarrow\downarrow\downarrow\uparrow\rangle$, where we assign $-\frac{1}{2}$ for $|\downarrow\rangle$ and $\frac{1}{2}$ for $|\uparrow\rangle$. When we consider a general matrix element $\langle\psi_2|\tilde{U}|\psi_1\rangle \propto \sum_{\mathbf{s}}\langle\psi_2|\cdots|\mathbf{s}\rangle\langle\mathbf{s}|\cdots|\psi_1\rangle$, δm for $|\psi_1\rangle, |\psi_2\rangle, |\mathbf{s}\rangle$ have the same even-odd parity. It is also clear that there are odd/even numbers of yellow gates for odd/even δm in the first half of the path ($\langle\mathbf{s}|\cdots|\psi_1\rangle$) and for odd/even $-\delta m$ in the second half of the path ($\langle\psi_2|\cdots|\mathbf{s}\rangle$). Since δm and $-\delta m$ have the same even-odd parity, the total number of the yellow gates in the path is even. Because the total number of all gates is even, the number of green gates is also even.

Owing to the even-time appearance of the green gates, the -1 phase under complex conjugation for the green gates cancels out for every path. Then we finally have

$$\langle\psi_2|V\tilde{U}V^\dagger|\psi_1\rangle = \langle\psi_2|\tilde{U}|\psi_1\rangle^* \quad (45)$$

for every matrix element, i.e., $\tilde{U}$ has the AUS. We note that, since $V^2 = 1$ for all T, $\tilde{U}$ belongs to symmetry Class AI and can have an exceptional point irrespective of T, in contrast with the case for the stroboscopic Ising model.

The symmetry structure for $J_3 = \pi/4$ can be discussed similarly: we

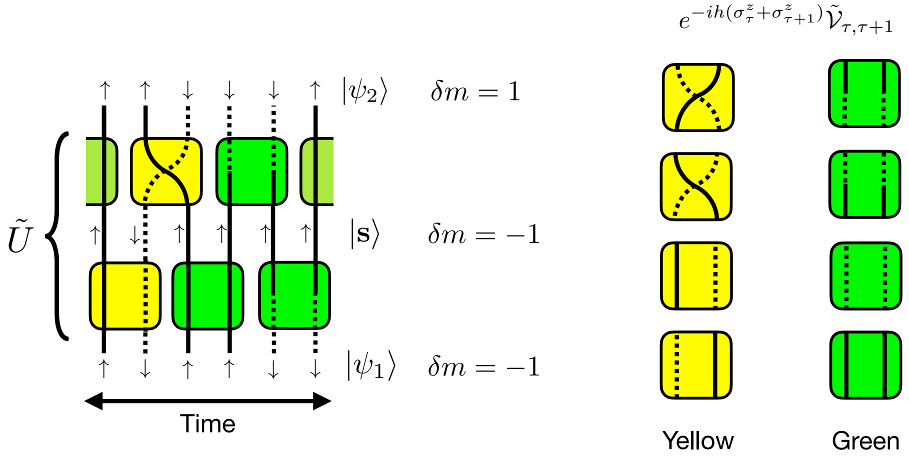

Supplementary Figure 10. **Example of a path of computational basis that constitutes a matrix element $\langle\psi_2|\tilde{U}|\psi_1\rangle$ of the spacetime-dual operator $\tilde{U}$.** We here consider $|\psi_1\rangle = |{\uparrow\downarrow\uparrow\uparrow\downarrow\downarrow}\rangle$ and $|\psi_2\rangle = |{\uparrow\uparrow\downarrow\downarrow\downarrow\uparrow}\rangle$, and insert a middle state $|\mathbf{s}\rangle = |{\uparrow\downarrow\uparrow\uparrow\uparrow\uparrow}\rangle$, where black lines denote $|{\uparrow}\rangle$ and dotted lines denote $|{\downarrow}\rangle$. These states are transformed by local two-site gates $e^{-ih(\sigma^z_\tau+\sigma^z_{\tau+1})}\tilde{\mathcal{V}}_{\tau,\tau+1}$, which are colored with yellow or green depending on the transformed spin states. By considering the difference of magnetization δm between even and odd sites, we can show that green and yellow gates must appear even times.

find that

$$V = \prod_{\tau:\text{odd}} e^{i\frac{\pi}{2}\sigma^x_\tau} \prod_{\tau:\text{even}} e^{i\frac{\pi}{2}\sigma^y_\tau} = i^T \prod_{\tau:\text{odd}} \sigma^x_\tau \prod_{\tau:\text{even}} \sigma^y_\tau \tag{46}$$

becomes AUS in this case. To see this, we note that

$$V\tilde{U}V^\dagger = \frac{1}{4} \prod_{\tau:\text{even}} V_2' e^{-ih(\sigma^z_\tau+\sigma^z_{\tau+1})} \tilde{\mathcal{V}}_{\tau,\tau+1} V_2'^\dagger \prod_{\tau:\text{odd}} V_2 e^{-ih(\sigma^z_\tau+\sigma^z_{\tau+1})} \tilde{\mathcal{V}}_{\tau,\tau+1} V_2^\dagger, \tag{47}$$

where V_2 and V_2' are shorthand notations for $\sigma^x_\tau \sigma^y_{\tau+1}$ and $\sigma^y_\tau \sigma^x_{\tau+1}$, respectively. Again, we can calculate nonzero matrix elements of local gates as

$$\begin{aligned} \langle \uparrow\downarrow | V_2 e^{-ih(\sigma^z_\tau+\sigma^z_{\tau+1})} \tilde{\mathcal{V}}_{\tau,\tau+1} V_2^\dagger | \uparrow\downarrow \rangle &= \langle \downarrow\uparrow | e^{-ih(\sigma^z_\tau+\sigma^z_{\tau+1})} \tilde{\mathcal{V}}_{\tau,\tau+1} | \downarrow\uparrow \rangle \\ &= i \langle \uparrow\downarrow | e^{-ih(\sigma^z_\tau+\sigma^z_{\tau+1})} \tilde{\mathcal{V}}_{\tau,\tau+1} | \uparrow\downarrow \rangle^* = -ie^{-\frac{\pi}{4}i} \sin J_- \end{aligned} \tag{48}$$

$$\begin{aligned} \langle \downarrow\uparrow | V_2 e^{-ih(\sigma^z_\tau+\sigma^z_{\tau+1})} \tilde{\mathcal{V}}_{\tau,\tau+1} V_2^\dagger | \uparrow\downarrow \rangle &= - \langle \uparrow\downarrow | e^{-ih(\sigma^z_\tau+\sigma^z_{\tau+1})} \tilde{\mathcal{V}}_{\tau,\tau+1} | \downarrow\uparrow \rangle \\ &= i \langle \downarrow\uparrow | e^{-ih(\sigma^z_\tau+\sigma^z_{\tau+1})} \tilde{\mathcal{V}}_{\tau,\tau+1} | \uparrow\downarrow \rangle^* = -e^{-\frac{\pi}{4}i} \sin J_+ \end{aligned} \tag{49}$$

$$\begin{aligned} \langle \downarrow\downarrow | V_2 e^{-ih(\sigma^z_\tau+\sigma^z_{\tau+1})} \tilde{\mathcal{V}}_{\tau,\tau+1} V_2^\dagger | \uparrow\uparrow \rangle &= - \langle \uparrow\uparrow | e^{-ih(\sigma^z_\tau+\sigma^z_{\tau+1})} \tilde{\mathcal{V}}_{\tau,\tau+1} | \downarrow\downarrow \rangle \\ &= -i \langle \downarrow\downarrow | e^{-ih(\sigma^z_\tau+\sigma^z_{\tau+1})} \tilde{\mathcal{V}}_{\tau,\tau+1} | \uparrow\uparrow \rangle^* = -ie^{-\frac{\pi}{4}i} \cos J_+ \end{aligned} \tag{50}$$

$$\begin{aligned} \langle \downarrow\downarrow | V_2 e^{-ih(\sigma^z_\tau+\sigma^z_{\tau+1})} \tilde{\mathcal{V}}_{\tau,\tau+1} V_2^\dagger | \downarrow\downarrow \rangle &= \langle \uparrow\uparrow | e^{-ih(\sigma^z_\tau+\sigma^z_{\tau+1})} \tilde{\mathcal{V}}_{\tau,\tau+1} | \uparrow\uparrow \rangle \\ &= -i \langle \downarrow\downarrow | e^{-ih(\sigma^z_\tau+\sigma^z_{\tau+1})} \tilde{\mathcal{V}}_{\tau,\tau+1} | \downarrow\downarrow \rangle^* = e^{-\frac{\pi}{4}i} e^{-2ih} \cos J_- \end{aligned} \tag{51}$$

$$\begin{aligned} \langle \uparrow\uparrow | V_2 e^{-ih(\sigma^z_\tau+\sigma^z_{\tau+1})} \tilde{\mathcal{V}}_{\tau,\tau+1} V_2^\dagger | \uparrow\uparrow \rangle &= \langle \downarrow\downarrow | e^{-ih(\sigma^z_\tau+\sigma^z_{\tau+1})} \tilde{\mathcal{V}}_{\tau,\tau+1} | \downarrow\downarrow \rangle \\ &= -i \langle \uparrow\uparrow | e^{-ih(\sigma^z_\tau+\sigma^z_{\tau+1})} \tilde{\mathcal{V}}_{\tau,\tau+1} | \uparrow\uparrow \rangle^* = e^{-\frac{\pi}{4}i} e^{2ih} \cos J_-, \end{aligned} \tag{52}$$

where $J_3 = \pi/4$ is used. Again, the two-site transitions for $\{|\uparrow\uparrow\rangle \to |\uparrow\uparrow\rangle, |\uparrow\uparrow\rangle \to |\downarrow\downarrow\rangle, |\downarrow\downarrow\rangle \to |\uparrow\uparrow\rangle, |\downarrow\downarrow\rangle \to |\downarrow\downarrow\rangle\}$ appear even times. Consequently, the complex conjugation operation leaves the overall factor i^T, i.e.,

$$\langle \psi_2 | V\tilde{U}V^\dagger | \psi_1 \rangle = (-1)^{T/2} \langle \psi_2 | \tilde{U} | \psi_1 \rangle^*, \tag{53}$$

meaning that $\tilde{U}$ has the AUS. We note that $VV^* = (-1)^{T/2}$ and thus $\tilde{U}$ belongs to Class AI/AII for even/odd $T/2$. Thus, the exceptional DQPT occurs only when $T/2$ is even in the case of $J_3 = \pi/4$.

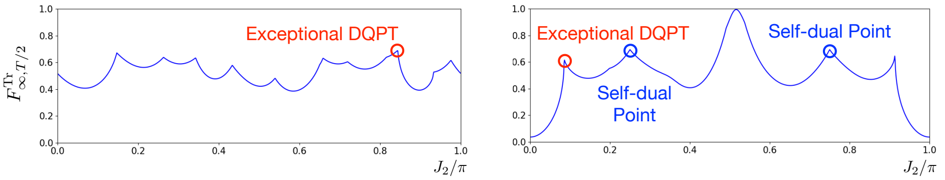

Supplementary Figure 11. **(Real part of) dynamical free energy $F^{\mathrm{Tr}}_{\infty,T/2}$ as a function of J_2.** We choose parameters as (left) $J_3 = \pi/2, J_1 = 0.15\pi, h = 1.0, T = 6$ and (right) $J_3 = \pi/4, J_1 = 0.47\pi, h = 1.0, T = 8$. As varying J_2, the exceptional dynamical quantum phase transition (DQPT) occurs for both of the cases, as well as DQPTs through self-dual points for $J_3 = \pi/4$.

Dynamical phase transitions

We demonstrate that the exceptional DQPT occurs for the above Floquet circuit model. In Supplementary Figure 11, we show dynamical free energy $F^{\mathrm{Tr}}_{\infty,T/2}$ for $J_3 = \pi/2$ and $J_3 = \pi/4$, where we vary J_2. We find that the exceptional DQPTs occur for both of the cases, thanks to the antiunitary symmetry hidden in the spacetime-dual operator $\tilde{U}$. We also note that, there appear DQPTs through the self-dual points [6] with $J_3 = \pi/4$ and $J_2 = \pi/4, 3\pi/4$, where $F^{\mathrm{Tr}}_{\infty,T/2}$ universally takes $\log 2$.

Supplementary References

[1] Akila, M., Waltner, D., Gutkin, B. & Guhr, T. Particle-time duality in the kicked ising spin chain. *Journal of Physics A: Mathematical and Theoretical* **49**, 375101 (2016).

[2] Bertini, B., Kos, P. & Prosen, T. Exact spectral form factor in a minimal model of many-body quantum chaos. *Phys. Rev. Lett.* **121**, 264101 (2018). URL `https://link.aps.org/doi/10.1103/PhysRevLett.121.264101`.

[3] Bertini, B., Kos, P. & Prosen, T. Entanglement spreading in a minimal model of maximal many-body quantum chaos. *Phys. Rev. X* **9**, 021033 (2019). URL `https://link.aps.org/doi/10.1103/PhysRevX.9.021033`.

[4] Kim, H., Ikeda, T. N. & Huse, D. A. Testing whether all eigenstates obey the eigenstate thermalization hypothesis. *Phys. Rev. E* **90**, 052105 (2014). URL `http://link.aps.org/doi/10.1103/PhysRevE.90.052105`.

[5] Zhang, J. *et al.* Observation of a many-body dynamical phase transition with a 53-qubit quantum simulator. *Nature* **551**, 601–604 (2017).

[6] Bertini, B., Kos, P. & Prosen, T. c. v. Exact correlation functions for dual-unitary lattice models in 1 + 1 dimensions. *Phys. Rev. Lett.* **123**, 210601 (2019). URL `https://link.aps.org/doi/10.1103/PhysRevLett.123.210601`.
